# The Conversion of 5,5′-Bi(1,2,3-dithiazolylidenes) into Isothiazolo[5,4-*d*]isothiazoles

**DOI:** 10.3390/molecules23061257

**Published:** 2018-05-24

**Authors:** Lidia S. Konstantinova, Ilia V. Baranovsky, Vlada V. Strunyasheva, Andreas S. Kalogirou, Vadim V. Popov, Konstantin A. Lyssenko, Panayiotis A. Koutentis, Oleg A. Rakitin

**Affiliations:** 1 N. D. Zelinsky Institute of Organic Chemistry, Russian Academy of Sciences, Moscow 119991, Russia; konstantinova_ls@mail.ru (L.S.K.); ilay679@rambler.ru (I.V.B.); vlada_0709@mail.ru (V.V.S.); 2 Nanotechnology Education and Research Center, South Ural State University, Chelyabinsk 454080, Russia; popov.ioc@gmail.com; 3 Department of Chemistry, University of Cyprus, P.O. Box 20537, Nicosia 1678, Cyprus; kalogirou.andreas@ucy.ac.cy; 4 Department of Life Sciences, School of Sciences, European University Cyprus, 6 Diogenis Str., Engomi, P.O. Box 22006, Nicosia 1516, Cyprus; 5 A. N. Nesmeyanov Institute of Organoelement Compounds, Russian Academy of Sciences, Moscow 119991, Russia; kostya@ineos.ac.ru

**Keywords:** sulfur-nitrogen heterocycles, dithiazoles, isothiazoles, ring transformation

## Abstract

Thermolysis of 4,4′-dichloro-, 4,4′-diaryl-, and 4,4′-di(thien-2-yl)-5,5′-bi(1,2,3-dithiazol-ylidenes) affords the respective 3,6-dichloro-, 3,6-diaryl- and 3,6-di(thien-2-yl)isothiazolo[5,4-*d*]-isothiazoles in low to high yields. The transformation of the 4,4′-diaryl- and 4,4′-di(thien-2-yl)-5,5′-bi(1,2,3-dithiazolylidenes) occurs at lower temperatures in the presence of the thiophiles triphenylphosphine or tetraethylammonium iodide. Optimized reaction conditions and a mechanistic rationale for the thiophile-mediated ring transformation are presented.

## 1. Introduction

**Figure 1:**
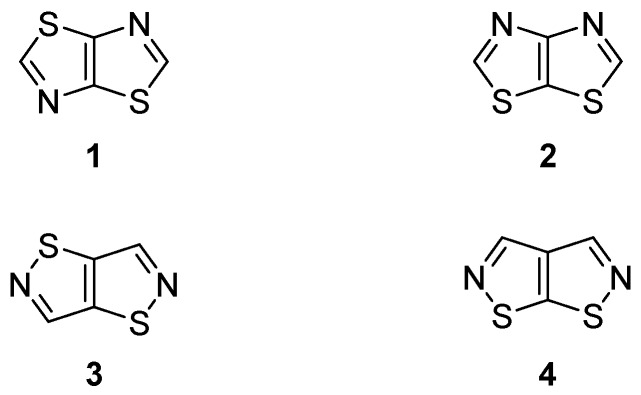
Known [1,2] and [1,3] thiazolothiazole ring systems **1**–**4**.

Thienothiophenes are rigid *π* rich arenes used to build biologically active compounds [1] and semi-conducting or fluorescent small molecules, oligomers and polymers [2,3]. Owing to their high HOMO energy levels, thienothiophenes are oxidatively unstable but this can be overcome by introducing electron withdrawing substituents, or by replacing a ring *sp*^2^ carbon with a more electronegative *sp*^2^ nitrogen. An example of the latter strategy is the replacement of thienothiophene with thiazolo[5,4-*d*]thiazole (**1**). Since the first thiazolo[5,4-*d*]thiazole (**1**) was reported in 1960 [4], over 400 analogues have been made and many were incorporated into dyes, oligomers and polymers for semiconductors and plastic electronics [5,6]. Interestingly, the isomeric thiazolo[4,5-*d*]thiazole (**2**) which, like thiazolo[5,4-*d*]thiazole (**1**), shares a common C-C bond between the two thiazoles is less well known; only 14 analogues are known [7,8,9,10,11,12,13]. Despite this, several analogues have useful properties as plant fungacides [10], antitumour agents [7], and as non-linear optical materials [8]. The isomeric isothiazole analogues are also poorly studied, which is surprising as they offer the possibility of fusion across more than one common C-C bond, affording up to six possible isothiazoloisothiazole biheterole structures. Of these, syntheses and chemistry of only two have been reported: isothiazolo[5,4-*d*]isothiazole (**3**) [14,15,16] and isothiazolo[4,5-*d*]-isothiazole (**4**) [17,18,19] (Figure 1).

**Scheme 1:**
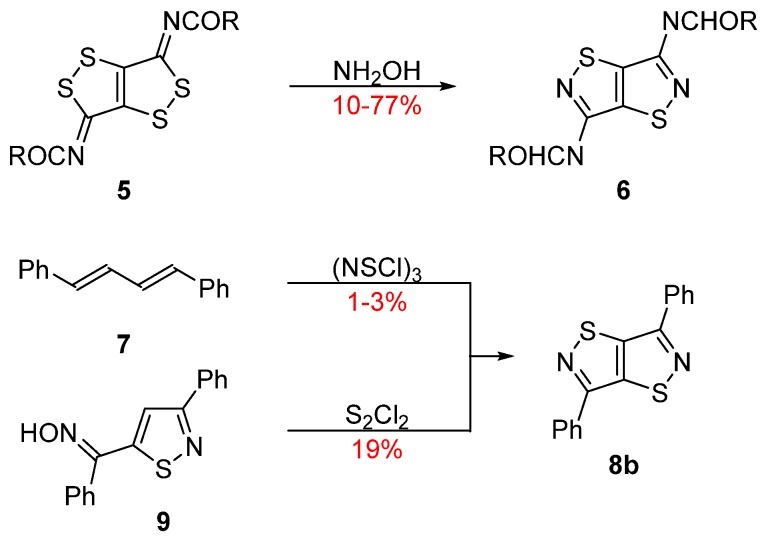
Known routes to isothiazolo[5,4-*d*]isothiazoles.

Three strategies are reported for the preparation of isothiazolo[5,4-*d*]isothiazoles: (1) 3,6-bis(acylimino)-3*H*,6*H*-[1,2]dithiolo[4,3-*c*][1,2]dithioles **5** react with hydroxylamine to undergo an Assisted Nucleophilic Ring Opening Ring Closure (ANRORC) style exchange of S for NH to give 3,6-bis(acylamino)isothiazolo[5,4-*d*]isothiazoles **6** [15]; (2) 1,4-diphenylbuta-1,3-diene (**7**) reacts with trithiazyl trichloride to afford 3,6-diphenylisothiazolo[5,4-*d*]isothiazole (**8b**) [14]; and (3) 5-benzoyl-3-phenylisothiazole oxime (**9**) reacts with disulfur dichloride in DMF at 100 °C for 16 h to give isothiazolo[5,4-*d*]isothiazole (**8b**) [14,16] (Scheme 1). Considering this and the potential uses of isothiazolo[5,4-*d*]isothiazoles like **3** in the materials sciences, we were interested in developing a new complementary route to this ring system.

**Scheme 2:**
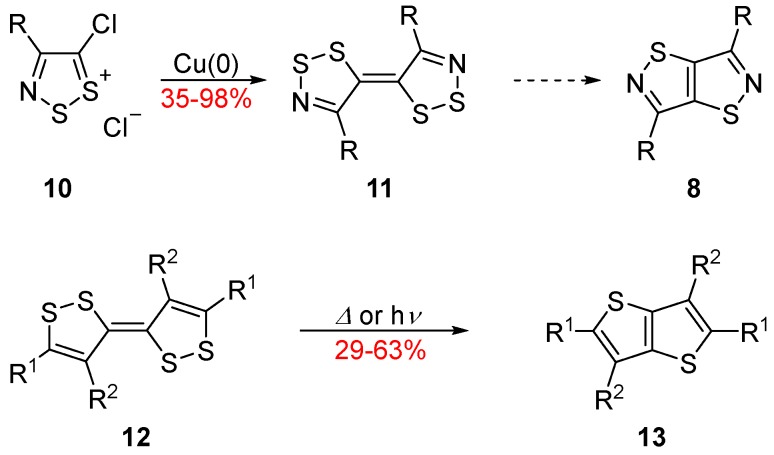
Ring transformation of (*E*)-3,3′-bi(1,2-dithiolylidenes) **12** into thieno[3,2-*b*]thiophenes **13** and the analogous proposed transformation of 5,5′-bi(1,2,3-dithiazolylidenes) **11** into isothiazolo[5,4-*d*]isothiazoles **8**.

Recently, Rakitin et al. described the Cu(0)-mediated coupling of 4-substituted 5-chloro-1,2,3-dithiazolium chlorides **10** [20,21,22] to give (*E*)-4,4′-disubstituted 5,5′-bi(1,2,3-dithiazolylidenes) **11** [23]. Products **11** are similar to (*E*)-3,3′-bi(1,2-dithiolylidenes) **12** that undergo both thermal and light-mediated ring transformations to afford thieno[3,2-*b*]thiophenes **13** [24] (Scheme 2).

The chemistry of 1,2,3-dithiazoles has been extensively reviewed [25,26,27] and 4-chloro-5*H*-1,2,3-dithiazoles undergo both thermal [28,29,30,31] and ANRORC-mediated [32,33,34] ring transformations to afford various heterocycles, including both thiazoles [28,29,30,31,35,36,37,38] and isothiazoles [36,39,40,41,42]. As such, we proposed that 5,5′-bi(1,2,3-dithiazolylidenes) **11** could be similarly converted into isothiazolo[5,4-*d*]isothiazoles **8** providing a new route to this rare biheterole.

## 2. Results and Discussion

**Scheme 3:**
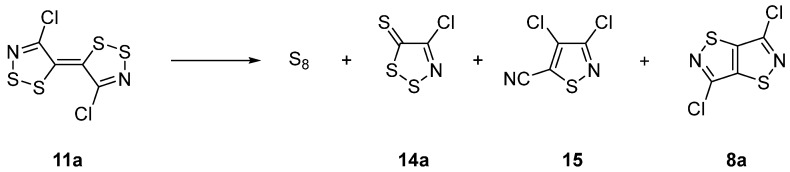
Decomposition of (*E*)-4,4′-dichloro-5,5′-bi(1,2,3-dithiazolylidene) (**11a**).

Early studies on (*E*)-4,4′-dichloro-5,5′-bi(1,2,3-dithiazolylidene) (**11a**) revealed the compound to be unstable. DCM solutions of dithiazolylidene **11a** in the presence of daylight slowly became complex (by TLC). Furthermore, samples of crystalline dithiazolylidene **11a** after several months also showed signs of decomposition. Chromatographic analysis of a decomposed sample revealed the presence of elemental sulfur (S_8_), 4-chloro-5*H*-1,2,3-dithiazole-5-thione (**14**) [43], 3,4-dichloro-isothiazole-5-carbonitrile (**15**) [39,44], a new compound identified as 3,6-dichloroisothiazolo- [5,4-*d*]isothiazole (**8a**) as well as several unstable (2D TLC), unidentified yellow and orange products (Scheme 3).

**Figure 2:**
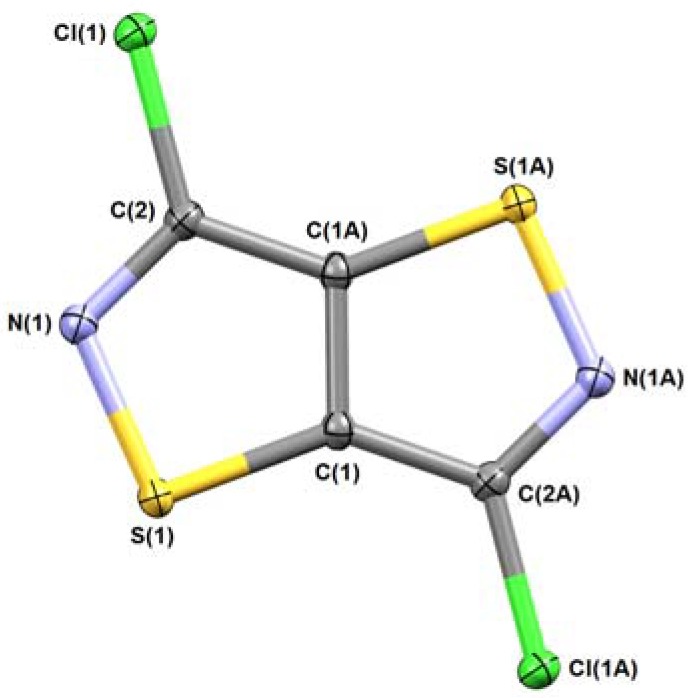
X-ray structure of 3,6-dichloroisothiazolo[5,4-*d*]isothiazole (**8a**). (CCDC 1840070). Thermal ellipsoids are at 50% probability.

To the best of our knowledge, 3,6-dichloroisothiazolo[5,4-*d*]isothiazole (**8a**) is a new dihaloisothiazoloisothiazole and a potentially useful biheterole building block. To support its structure, single crystal XRD crystallography was carried out (Figure 2).

Interestingly, thermolysis of a neat sample of freshly prepared (*E*)-4,4′-dichloro-5,5′-bi(1,2,3-dithiazolylidene) (**11a**) (0.10 mmol) at ca. 300 °C under argon atmosphere led to the formation of the dichloroisothiazoloisothiazole **8a** in a low yield (8%) together with elemental sulfur (S_8_) (TLC) (Table 1, entry 1). Fortunately, thermolysis of 4,4′-di(het)aryl-substituted bi(dithiazolylidenes) **11b**–**f** provided the corresponding 3,6-di(het)arylisothiazolo[5,4-*d*]isothiazoles **8b**–**f** in 82–96% yields (Table 1, entries 2–6).

Unable to scale up the thermolysis for the conversion of (*E*)-4,4′-dichloro-5,5′-bi(1,2,3-dithiazolylidene) **11a** into the isothiazolo[5,4-*d*]isothiazole **8a** we then investigated the use of thiophilic agents triphenylphosphine, BnEt_3_NCl or Et_4_NI, but obtained in each case only an intractable tarry mass. Not surprisingly, dichloroisothiazoloisothiazole **8a**, which hosts a variety of highly electrophilic sites (S, Cl, C3/6 as well as C3a/6a) was unstable to the thiophiles. This was partially attributed to the excellent nucleofuge ability of the C3/6 chlorine substituents. This lability was also evident during efforts to carry out substitution of the chlorides by methoxide or pyrrolidine nucleophiles, the former leading to intractable baseline and the latter to a mixture of monocyclic oligosulfides (TLC, NMR) that could not be isolated pure. Moreover our efforts to carry out Suzuki-Miyaura [PhB(OH)_2_ (3 equiv), KF (3.5 equiv), 18-crown-6 (0.5 equiv), Pd(OAc)_2_ (10 mol %), PhMe, 110 °C], Stille [PhSnBu_3_ (3 equiv), Pd(Oac)_2_ (10 mol %), DMF, 100 °C or Pd Superstable (10 mol %), PhMe, 110 °C] or Sonogashira couplings [phenylacetylene (2.2 equiv), Et_3_N (4 equiv), PdCl_2_(Ph_3_P)_2_ (5 mol %), MeCN, 100 °C] led to only traces of product and mainly gave degradation of the ring system. The difficulty in displacing isothiazole C3 chlorides has been previously reported [45,46,47]. Analysis of the reaction mixtures (TLC) revealed S_8_ (TLC) supporting ring cleavage, presumably owing to a thiophilic attack.

**Figure 3:**
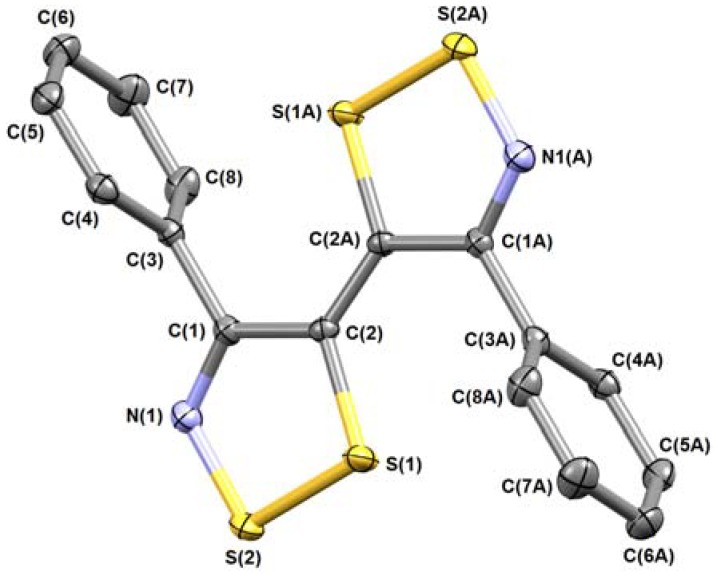

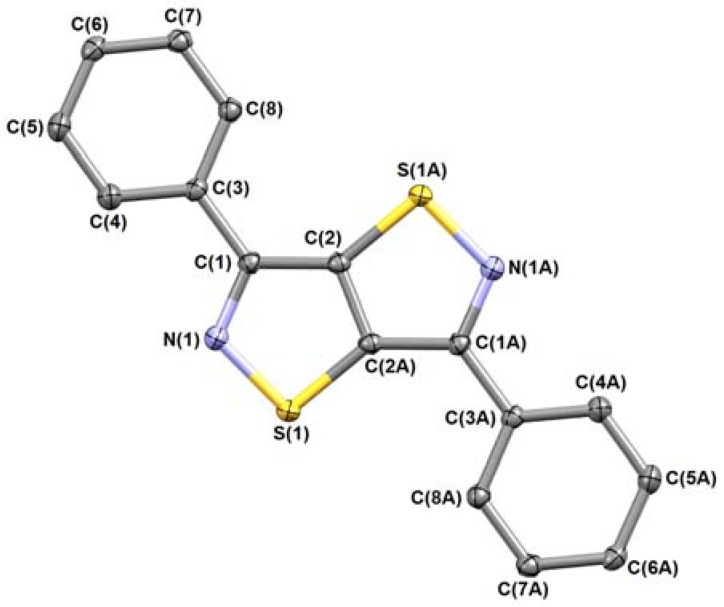
X-ray structures of (*E*)-4,4′-diphenyl-5,5′-bi(1,2,3-dithiazolylidene) (**11b**) (CCDC 1840072) (**top**) and 3,6-diphenylisothiazolo[5,4-*d*]isothiazole (**8b**) (CCDC 1840071) (**bottom**). Thermal ellipsoids are at 50% probability and hydrogens are omitted for clarity.

In light of the above, and with the aim to develop a milder route to 3,6-di(het)aryl-isothiazolo[5,4-*d*]isothiazoles **11** we developed and optimized a thiophile-mediated ring transformation for (*E*)-4,4′-diphenyl-5,5′-bi(1,2,3-dithiazolylidene) (**11b**), which has no nucleofuges at either C4/4′ and gives a product with no nucleofuges at either C3/6 and therefore was more resistant to thiophile-mediated ring opening reactions (Table 2, entries 1–10). Interestingly, single crystal X-ray studies were also obtained to support both the (*E*)-geometry of the diphenyl- bi(dithiazolylidene) **11b** and the structure of the final diphenyl-substituted isothiazoloisothiazole **8b** (Figure 3). While both isothiazoloisothiazoles **8a** and **8b** have planar isothiazoloisothiazole core structures, the phenyl groups in the latter deviate in a conrotatory manner from the isothiazoloisothiazole plane by 8.7°.

Heating a toluene solution of the diphenylbi(dithiazolylidene) **11b** at ca. 110 °C for 29 h led to its conversion into diphenylisothiazoloisothiazole **8b** in 76% yield together with traces of S_8_ and 4-phenyl-5*H*-1,2,3-dithiazole-5-thione (**14b**) [20] (by TLC) (Table 2, entry 1). In the presence of thiophiles Et_4_NI or Ph_3_P the reaction proceeded significantly faster and in higher yield (Table 2, entries 2–10). When Ph_3_P (2–4 equiv) was used as thiophile, diphenylisothiazoloisothiazole **8b** was formed in quantitative yield but was accompanied, as expected, by the formation of triphenylphosphine sulfide (Ph_3_P = S) in 75–78% yield (Table 2, entries 2 and 3), which required chromatographic separation. Fortunately, the use of Et_4_NI as thiophile worked equally well, and on a 0.5 mmol scale, in 5 mL of PhMe, the use of Et_4_NI (0.2 equiv) gave the fastest reaction (2 h) and a quantitative yield of product (Table 2, entry 5) which could be isolated without the need for chromatography. More or less equivalents of Et_4_NI led to longer reaction times (Table 2, entries 4 and 6). With these conditions in hand, we then investigated the effect of concentration and temperature. Fortunately, small variations in concentration did not affect the reaction times or yields (data not shown), and carrying out the reaction using 0.1 mmol of ylidene **11b** in only 5 mL of PhMe continued to give a near quantitative yield of isothiazoloisothiazole **8b** (95%) together with some dithiazolethione **14b** (5%) (Table 2, entry 9), but at 0.2 mmol the yield of the desired product **8b** dropped significantly (63%) and the amount of undesired dithiazolethione **14b** increased (26%) (Table 2, entry 10). Lowering the reaction temperature with the use of PhH (bp 80 °C) instead of PhMe (bp 110 °C) led to no reaction after 10 h of heating (Table 2, entry 7) while the use of PhCl (bp 132 °C) led to a longer reaction time (8 h), lower yield (72%) and the formation of more dithiazolethione **14b** (15%) compared to the PhMe (Table 2, entry 8). With the thiophile-mediated reaction of the diphenylbi(dithiazolylidene) **11b** partially optimized we then carried out a minor investigation into the reactions scope (Table 2, entries 11–13).

Under the optimized reaction conditions, ylidenes bearing aryls containing electron withdrawing *para*-fluoro and electron releasing *para*-methoxy substitution worked to give the desired isothiazoloisothiazoles in moderate to excellent yields 69 and 99%, respectively (Table 2, entries 11 and 12). Furthermore, the important thien-2-yl group, an electron rich hetaryl that is important in organic electronic materials, was tolerated to afford 3,6-di(thien-2-yl)-isothiazolo[5,4-*d*]isothiazole (**8e**) in 92% yield (Table 2, entry 13), potentially opening up this biheterole system for study as a new *π* spacer for small organic molecules, oligomers or polymers in material sciences.

**Scheme 4:**
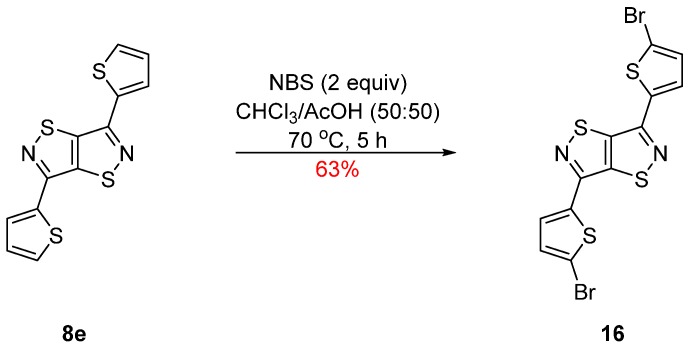
Preparation of 3,6-di(5,5′-dibromothien-2-yl)isothiazolo[5,4-*d*]isothiazole (**16**).

Interestingly, treating di(thien-2-yl)isothiazoloisothiazole **8e** with *N*-bromosuccinimide (NBS) (2 equiv) in a mix of chloroform/acetic acid (50:50) heated at ca. 70 °C for 5 h afforded the useful 3,6-di(5,5′-dibromothien-2-yl)isothiazolo[5,4-*d*]isothiazole (**16**) in 63% yield (Scheme 4). Efforts to incorporate this moiety in oligomers and polymers for organic electronic applications are now in progress.

### Mechanistic Rationale

**Scheme 5:**
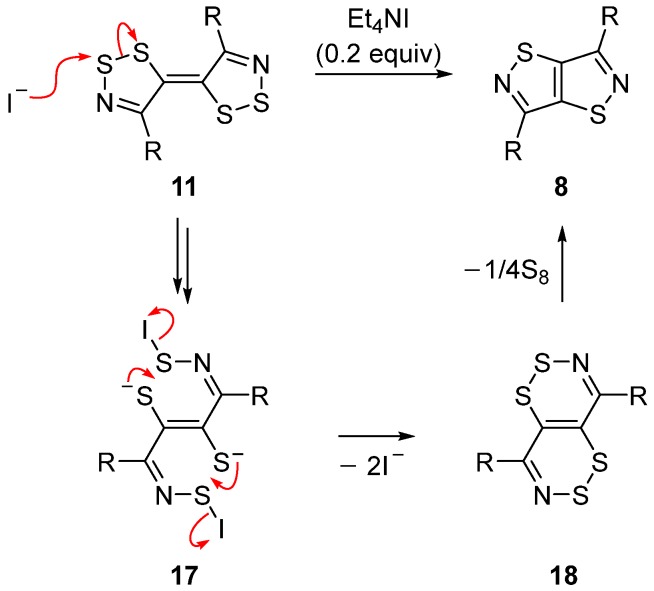
Tentative mechanism for the Et_4_NI-mediated ring transformation of dithiazolylidenes **11** into isothiazolo[5,4-*d*]isothiazoles **8**.

Tentatively, we propose the thiophile-mediated reaction (Table 2) proceeded via an ANRORC style reaction pathway [32,33,34], where the thiophile attacks either the dithiazole S1 or S2 sulfurs to generate a ring opened species that then collapses to give the isothiazole ring system (Scheme 5). At this stage, it is not possible to give an accurate mechanism, as several possibilities exist. Previous studies, nevertheless, reveal the dithiazole S2 atom to be marginally more susceptible to thiophilic attack [49], and based on this we propose the ring opening of both dithiazoles to generate the dianion **17** that then collapses to the 14*π* 4,8-disubstituted [1–3]dithiazino[6,5-*e*][1,2,3]dithiazine **18** which collapses to the thermodynamically more stable 10*π* 3,6-disubstituted isothiazolo-[5,4-*d*]isothiazole **8** (Scheme 5). Attempts to treat 3,6-diphenylisothiazolo[5,4-*d*]isothiazole **8b** with elemental sulfur or active sulfur (DABCO/S_8_) [50] led to no reaction supporting that the reaction was not in equilibrium and the product was not convertible back to the starting dithiazole **11b**.

Similar ANRORC-style transformations of bis(1,2,3-dithiazoles) have been reported that afford difficult to access 1,3,4-thiadiazoles and thiazoles [37]. These transformations involved thiophile-mediated ring opening of one dithiazole to release a nucleophilic sulfur that was then intramolecularly trapped by the neighboring dithiazole to generate a new more thermodynamically stable heteroarene. More recently, evidence for the conversion of 1,2,3-dithiazoles into structurally related 1,2,4-dithiazines has also been reported [51].

The mechanistic rationale for the thermolysis of neat samples or for the decomposition of samples on prolonged storage or in solution, remains unclear and, similar to the ring transformation of (*E*)-3,3′-bi(1,2-dithiolylidene) to thieno[3,2-*b*]thiophenes could also involve homolytic pathways [24]. In the presence of spin trap agents such as 1,4-benzoquinone or (2,2,6,6-tetramethyl-piperidin-1-yl)oxyl oxidanyl (TEMPO) we noted that the ring transformation was significantly slower.

## 3. Materials and Methods

### 3.1. General Methods and Materials

Powdered anhydrous Na_2_SO_4_ was used for drying organic extracts and all volatiles were removed under reduced pressure. Toluene was distilled over CaH_2_ before use. All reaction mixtures and column eluents were monitored by TLC using commercial glass backed thin layer chromatography (TLC) plates (Merck Kieselgel 60 F_254_). The plates were observed under UV light at 254 and 365 nm. Elemental analyses were performed on a 2400 Elemental Analyzer (Perkin Elmer Inc., Waltham, MA, USA). Melting points were determined on a Kofler hot-stage apparatus and are uncorrected. Solvents used for recrystallization are indicated after the melting point. ^1^H and ^13^C-NMR spectra were taken with a Bruker AM-300 (at 300.1 and 75.5 MHz) or Bruker DRX500 (at 500.1 and 125.8 MHz) or Bruker AV600 instrument (at 600.1 and 150.9 MHz) (Bruker Ltd., Moscow, Russia) with TMS as the standard. *J* values are given in Hz. MS spectra (EI, 70 eV) were obtained with a MAT INCOS 50 instrument (Thermo Finnigan LLC, San Jose, CA, USA). High-resolution MS spectra were measured on a Bruker MICROTOF II instrument using electrospray ionization (ESI). The measurement was operated in a positive ion mode (interface capillary voltage—4500 V) or in a negative ion mode (3200 V); mass range was from *m/z* 50 to 3000 Da; external or internal calibration was done with Electrospray Calibrant Solution (Fluka Chemicals Ltd., Gillingham, UK). A syringe injection was used for solutions in acetonitrile, methanol, or water (flow rate 3 μL/min). Nitrogen was applied as a dry gas; interface temperature was set at 180 °C. IR spectra were measured with a M-80 instrument (Carl Zeiss Jena GmbH, Jena, Germany) in KBr pellets. 4,4′-Disubstituted 5,5′-bi(1,2,3-dithiazolylidenes) **11a**–**e** [23] and (*E*)-1-(4-bromophenyl)-ethan-1-one oxime [52] were prepared according to the literature.

Data collection for a single crystals **8a**, **8b**, **8d** and **11b** (Figures S1–S4, Supporting Information, SI) was performed at the Center for Molecular Composition Studies of INEOS RAS on a Bruker Smart Apex II CCD diffractometer (Mo K*α* radiation, *λ* = 0.71073 Å, graphite monochromator). Frames were integrated using the Bruker SAINT software package [53] using a narrow-frame algorithm, and a semiempirical absorption correction was applied with the SADABS program [54] using intensity data of the equivalent reflections. All the structures were solved by direct method and refined by the least-squares in anisotropic full-matrix approximation on *F*^2^_hkl_. The hydrogen atoms were calculated geometrically and refined in isotropic approximation using the riding model with the SHELX software package [55]. The refinement of the molecules with minor occupancy was performed with the restraints on anisotropic displacement parameters (EADP) and bond lengths and angles (SAME). Detailed crystallographic information is provided in Table 3 and as Supporting Information in CIF format that can be obtained free of charge via http://www.ccdc.cam.ac.uk/conts/retrieving.html or from the Cambridge Crystallographic Data Centre, 12, Union Road, Cambridge CB2 1EZ, UK; Fax: 44-1223-336033 using the reference CCDC numbers (Table 3).

### 3.2. (E)-4,4′-Bis(4-bromophenyl)-5,5′-Bi(1,2,3-dithiazolylidene) (**11f**)

To a stirred solution of (*E*)-1-(4-bromophenyl)ethan-1-one oxime [24] (428 mg, 2 mmol) and sulfur monochloride (0.64 mL, 4 mmol) in acetonitrile (15 mL) at ca. −5 °C under an argon atmosphere was added dropwise pyridine (0.96 mL, 6 mmol). The mixture was stirred at ca. −5 °C for 15 min, then copper powder (192 mg, 3 mmol) was added, the mixture was stirred at room temperature for 1.5 h and then poured into ice water (100 mL). The precipitate was filtered, washed with water, dried and extracted with CH_2_Cl_2_ (3 × 15 mL). The combined extracts were dried (CaCl_2_) and solvents were evaporated under reduced pressure. The residue was rapidly separated by flash chromatography (silica gel Merck 60, *n*-hexane and then *n*-hexane/CH_2_Cl_2_ mixtures) to afford the title compound **11f** (346 mg, 67%) as a black powder, m.p. 181–182 °C (*n*-hexane); *δ*_H_ (300 MHz; СD_2_Cl_2_) 7.37 (d, 4H, *J* 6.6, Ar *H*), 7.67 (d, 4H, *J* 8.1, Ar *H*); *δ*_C_ (75 MHz; СD_2_Cl_2_) 157.1, 150.6, 132.7, 130.8, 127.6, 110.1; *ν*_max_ (KBr) 3088, 3067, 3043, 1671, 1482, 1459, 1392, 1322, 1262, 1100, 1072, 1025, 1010, 827, 804, 758, 677 cm^−1^; *m/z* (EI) 518 (M^+^ + 4, 53%), 516 (M^+^ + 2, 100), 514 (M^+^, 46), 484 (24), 452 (68), 292 (3), 258 (3), 102 (9), 88 (10); HRMS *m/z* (ESI) 515.7903 [M]^+^ (calc. for C_16_H_8_Br_2_N_2_S_4_, *m/z* 515.7910).

### 3.3. General Thermolysis Procedure (Method 1)

4,4′-Disubstituted 5,5′-bi(1,2,3-dithiazolylidene) **11** (0.10 mmol) under an argon atmosphere was immersed into a preheated (~230–300 °C) Wood′s metal bath for 0.5 min. On cooling to ca. 20 °C the residue was triturated with *n*-hexane, filtered and recrystallized to give isothiazolo[5,4-*d*]-isothiazole **8** as colorless crystals (see Table 1).

### 3.4. General ANRORC Procedure (Method 2)

To a stirred solution of 4,4′-disubstituted 5,5′-bi(1,2,3-dithiazolylidene) **8** (0.05 mmol) in anhydrous toluene (5 mL) at ca. 20 °C was added Et_4_NI (0.2 equiv). The reaction mixture was then heated at reflux (ca. 110 °C) until complete consumption of the starting dithiazole **8** (by TLC). The reaction mixture was allowed to cool to ca. 20 °C, the volatiles removed in vacuo, and then the residue was triturated with *n*-hexane (1.5 mL), the precipitate was filtered and washed (acetone 2 × 1.5 mL) and dried. Recrystallisation of the residue gave the isothiazolo[5,4-*d*]isothiazole **8** as colorless crystals (see Table 2).

### 3.5. Data on Compounds ***8a**–**f***

#### 3.5.1. 3,6-Dichloroisothiazolo[5,4-*d*]isothiazole (**8a**)

(Method 1: 2 mg, 8%) as colorless prisms, m.p. 210–212 °C (decomp.) (acetone). (Found: C, 22.98, N, 13.01. C_4_Cl_2_N_2_S_2_ requires C, 22.76; N, 13.27%); *δ*_C_ (300 MHz; CDCl_3_) 155.9, 138.4; *ν*_max_ (KBr) 1596, 1332, 1188, 1076, 792, 768 cm^−1^; *λ*_max_ (CH_2_Cl_2_)/nm 236 (log *ε* 3.31), 304 (3.98); *m/z* (EI) 214 (M^+^ + 4, 15%), 212 (M^+^ + 2, 63), 210 (M^+^, 95), 175 (11), 114 (22), 88 (50), 70 (100).

#### 3.5.2. 3,6-Diphenylisothiazolo[5,4-*d*]isothiazole (**8b**)

(Method 1: 28.0 mg, 96%; Method 2: 14.6 mg, 99%) as colorless blocks, m.p. 203–204 °C (lit. [14], m.p. 200–202 °C) (acetone); (found: C, 65.43; H, 3.56; N, 9.25. C_16_H_10_N_2_S_2_ requires: C, 65.28; H, 3.42; N, 9.52%); *δ*_H_ (300 MHz; CDCl_3_) 8.00 (d, 4H, *J* 7.0, Ar *H*), 7.59–7.48 (m, 6H, Ar H); *δ*_C_ (75 MHz; CDCl_3_) 156.3, 156.1, 132.9, 130.2, 129.3, 127.3; *ν*_max_ (KBr) 1620, 1420, 1330, 1280, 1160, 978, 802, 763, 692 cm^−1^; *λ*_max_ (CH_2_Cl_2_)/nm 242 (log *ε* 4.59), 332 (4.32); *m/z* (EI) 294 (M^+^, 100%), 191 (77), 146 (37), 88 (100); HRMS *m*/*z* (ESI) 295.0336 [M + Na]^+^ (calc. for C_16_H_10_NaN_2_S_2_, *m*/*z* 295.0334).

#### 3.5.3. 3,6-Bis(4-fluorophenyl)isothiazolo[5,4-*d*]isothiazole (**8c**)

(Method 1: 27.4 mg, 83%; Method 2: 11.4 mg, 69%), as colorless crystals, m.p. 268–269 °C (acetone); (found: C, 58.33; H, 2.62; N, 8.23. C_16_H_8_F_2_N_2_S_2_ requires: C, 58.17; H, 2.44; N, 8.48%); *δ*_H_ (300 MHz; DMSO-*d*_6_) 8.06 (dd, 4H, *J* 4.8, Ar *H*), 7.49 (t, 4H, *J* 8.4, Ar *H*); *δ*_C_ (150 MHz; DMSO-*d*_6_) 165.4 (d, ^1^*J*_CF_ 226.5), 157.2, 155.9, 130.7 (d, ^3^*J*_CF_ 7.2), 130.1, 117.9 (d, ^2^*J*_CF_ 21.5); *ν*_max_ (KBr) 1600, 1515, 1440, 1420, 1315, 1238, 1180, 1100, 980, 840, 805, 720 cm^−1^; *λ*_max_ (CH_2_Cl_2_)/nm 242 (log *ε* 4.05), 330 (3.76); *m/z* (EI) 330 (M^+^, 50%), 235 (8), 209 (32), 164 (30), 121 (35), 88 (100); HRMS *m*/*z* (ESI) 330.0091 [M]^+^ (calc. for C_16_H_8_F_2_N_2_S_2_, *m*/*z* 330.0098).

#### 3.5.4. 3,6-Bis(4-methoxyphenyl)isothiazolo[5,4-*d*]isothiazole (**8d**)

(Method 1: 30.0 mg, 85%; Method 2: 17.6 mg, 99%), as colorless prisms, m.p. 238–239 °C (acetone); (found: C, 61.13; H, 4.15; N, 7.71. C_16_H_14_N_2_O_2_S_2_ requires: C, 60.99; H, 3.98; N, 7.90%); *δ*_H_ (300 MHz; DMSO-*d*_6_) 7.92 (d, 4H, *J* 8.1, Ar *H*), 7.22 (d, 4H, *J* 8.8, Ar *H*), 3.89 (s, 6H, OC*H*_3_); *δ*_C_ (150 MHz; DMSO-*d*_6_) 162.4, 157.7, 156.7, 129.8, 126.4, 116.3, 56.8 (OMe); *ν*_max_ (KBr) 1608, 1576, 1532, 1416, 1312, 1296, 1268, 1176, 1024, 832, 800 cm^−1^; *λ*_max_ (CH_2_Cl_2_)/nm 249 (log *ε* 4.35), 344 (4.17); *m/z* (EI) 354 (M^+^, 45%), 311 (2), 221 (22), 177 (27), 134 (30), 88 (100); HRMS *m*/*z* (ESI) 355.0546 [M + H]^+^ (calc. for C_18_H_15_N_2_O_2_S_2_, *m*/*z* 355.0546).

#### 3.5.5. 3,6-Di(thien-2-yl)isothiazolo[5,4-*d*]isothiazole (**8e**)

(Method 1: 28.0 mg, 90%; Method 2: 14.1 mg, 92%), as colorless crystals, m.p. 250–251 °C (acetone); (found: C, 47.26; H, 2.13; N, 8.93. C_12_H_6_N_2_S_4_ requires: C, 47.03; H, 1.97; N, 9.14%); *δ*_H_ (300 MHz; CDCl_3_) 7.52–7.48 (m, 4H, thienyl *H*), 7.21–7.18 (m, 2H, thienyl *H*); *δ*_C_ (75 MHz; CDCl_3_) 155.3, 150.9, 136.7, 129.0, 128.2, 127.7; *ν*_max_ (KBr) 3095 and 3031 (aryl С-H), 1652, 1536, 1448, 1424, 1344, 1256, 1224, 1048, 852, 800, 760, 704, 680, 664, 624 cm^−1^; *λ*_max_ (CH_2_Cl_2_)/nm 257 (log *ε* 4.08), 348 (3.98); *m/z* (EI) 306 (M^+^, 87%), 223 (3), 197 (20), 165 (9), 153 (20), 109 (25), 88 (100); HRMS *m*/*z* (ESI) 306.9463 [M + H]^+^ (calc. for C_12_H_7_N_2_S_4_, *m*/*z* 306.9463).

#### 3.5.6. 3,6-Bis(4-bromophenyl)isothiazolo[5,4-*d*]isothiazole (**8f**)

(Method 1: 30.0 mg, 82%), as colorless crystals, m.p. 234–235 °C (acetone); (found: C, 42.35; H, 1.59; N, 6.45. C_16_H_8_Br_2_N_2_S_2_ requires: C, 42.50; H, 1.78; N, 6.20%); *δ*_H_ (300 MHz; СD_2_Cl_2_) 7.93 (d, 4H, *J* 9.0, Ar *H*), 7.76 (d, 4H, *J* 9.0, Ar *H*); *δ*_C_ (125 MHz; СD_2_Cl_2_) 157.5, 154.7, 132.6, 131.7, 128.9, 122.7; *ν*_max_ (KBr) 1588, 1429, 1401, 1322, 1289, 1184, 1075, 1008, 972, 830, 805, 710, 652 cm^−1^; *λ*_max_ (CH_2_Cl_2_)/nm 254 (log *ε* 4.77), 337 (4.44); *m/z* (EI) 454 (M^+^ + 4, 57%), 452 (M^+^ + 2, 100), 450 (M^+^, 51), 295 (5), 271 (17), 190 (43), 155 (17), 102 (32), 88 (75); HRMS *m/z* (ESI) 452.8541 [M + H]^+^ (calc. for C_16_H_9_Br_2_N_2_S_2_, *m/z* 452.8548).

### 3.6. Bromination of 3,6-Di(thien-2-yl)isothiazolo[5,4-d]isothiazole *(**16**)*

#### 3,6-Di(5,5′-dibromothien-2-yl)isothiazolo[5,4-*d*]isothiazole (**16**)

To a stirred solution of 3,6-di(thien-2-yl)isothiazolo[5,4-*d*]isothiazole (**8f**) (73.4 mg, 0.24 mmol) in CHCl_3_/AcOH (50:50) (12 mL) at ca. 20 °C was added NBS (85.0 mg, 0.48 mmol). The mixture was then heated to ca. 70 °C for 7 h then allowed to cool to ca. 20 °C. The precipitate was filtered, washed with diethyl ether (2 × 2 mL), dried to afford the *title compound* **16** (34 mg, 31%) as colorless crystals. The combined solvents were evaporated under reduced pressure and separated by column chromatography on silica gel to afford addition quantity of the title compound **16** (36 mg, 32%). Combined yield of 3,6-di(5,5′-dibromothien-2-yl)isothiazolo[5,4-*d*]isothiazole (**16**) (70.0 mg, 63%); m.p. 255–257 °C (acetone); (found: C, 31.28; H, 1.03; N, 6.25. C_12_H_4_Br_2_N_2_S_4_ requires: C, 31.05; H, 0.87; N, 6.03%); *δ*_H_(300 MHz; D_2_SO_4_) 6.25 (d, 2H, *J* 4.4, thienyl *H*), 6.06 (d, 2H, *J* 4.4, thienyl *H*); *δ*_C_ (125 MHz; D_2_SO_4_) 150.0, 141.9, 137.2, 133.8, 131.1, 125.6; *ν*_max_ (KBr) 3091, 3051, 1656, 1598, 1543, 1452, 1398, 1349, 1299, 1212, 1120, 976, 934, 805, 782, 723, 660 cm^−1^; *λ*_max_ (CH_2_Cl_2_)/nm 279 (log *ε* 4.32), 354 (4.32); *m/z* (EI) 466 (M^+^ + 4, 60%), 464 (M^+^ + 2, 100), 462 (M^+^, 46), 384 (63), 306 (12), 222 (6), 88 (25); HRMS *m/z* (ESI) 464.7670 (MH)^+^ (calc. for C_12_H_5_Br_2_N_2_S_4_, *m/z* 464.7675).

## 4. Conclusions

4,4′-Dichloro- and 4,4′-di(het)aryl-5,5′-bi(1,2,3-dithiazolylidenes) undergo thermolysis to afford the respective 3,6-dichloro- and 3,6-di(het)arylisothiazolo[5,4-*d*]isothiazoles in low to high yields. The transformation of the di(het)arylbi(dithiazolylidenes) into di(het)arylisothiazoloisothiazoles can be achieved under milder conditions via thiophile-mediated ANRORC-type reactions with the use of Et_4_NI (0.2 equiv) as thiophile. The transformation provides access to 3,6-di(thien-2-yl)-substituted isothiazolo[5,4-*d*]isothiazole which can be valued scaffolds in molecular electronic materials.

## Figures and Tables

**Table 1:** Thermolysis of neat 4,4′-disubstitued 5,5′-bi(1,2,3-dithiazolylidenes) **11a**–**f** (0.10 mmol) for 2 min under argon to give 3,6-disubstituted isothiazolo[5,4-*d*]isothiazoles **8a**–**f**.

Entry	R	Temp (°C)	Yield 8 (%)
1	Cl	300	**8a** (8)
2	Ph	230	**8b** (96)
3	4-FC_6_H_4_	250	**8c** (83)
4	4-MeOC_6_H_4_	280	**8d** (85)
5	thien-2-yl	250	**8e** (90)
6	4-BrC_6_H_4_	270	**8f** (82)

**Table 2:**
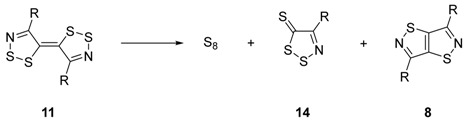
Optimization of the ring transformation of (*E*)-4,4′-diphenyl-5,5′-bi(1,2,3-dithiazolylidene) **11b** into 3,6-diphenylisothiazolo[5,4-*d*]isothiazole **8b** and scope of the reaction.

Entry	R	mmol	Thiophile	Solvent	Time	Yield 8	Yields Ph_3_P = S or 14
			(Equiv)	(mL)	(h)	(%)	(%)
1	Ph	0.05	—	PhMe (5)	29	**8b** (76)	
2	Ph	0.05	Ph_3_P (2)	PhMe (5)	3	**8b** (81)	Ph_3_P = S (94)
3	Ph	0.05	Ph_3_P (4)	PhMe (5)	2.5	**8b** (82)	Ph_3_P = S (96)
4	Ph	0.05	Et_4_NI (0.1)	PhMe (5)	17	**8b** (78)	
5	Ph	0.05	Et_4_NI (0.2)	PhMe (5)	2	**8b** (99) *^a^*	
6	Ph	0.05	Et_4_NI (2)	PhMe (5)	4	**8b** (98) *^a^*	
7	Ph	0.05	Et_4_NI (0.2)	PhH (5)	10	**8b** (nr) *^b^*	
8	Ph	0.05	Et_4_NI (0.2)	PhCl (5)	8	**8b** (72)	**14b** (15) [23]
9	Ph	0.10	Et_4_NI (0.2)	PhMe (5)	6	**8b** (95)	**14b** (5) [23]
10	Ph	0.20	Et_4_NI (0.2)	PhMe (5)	13	**8b** (63)	**14b** (26) [23]
11	4-FC_6_H_4_	0.05	Et_4_NI (0.2)	PhMe (5)	8	**8c** (69)	**14c** (9) [20]
12	4-MeOC_6_H_4_	0.05	Et_4_NI (0.2)	PhMe (5)	6	**8d** (99)	
13	thien-2-yl	0.05	Et_4_NI (0.2)	PhMe (5)	7	**8e** (92)	**14e** (1) [48]

*^a^* Chromatography free, trace of elemental sulfur by TLC. *^b^* NR = no reaction.

**Table 3:** Crystallographic data for **8a**, **8b**, **8d** and **11b**.

	8a	8b	8d	11b
CCDC	1840070	1840071	1840073	1840072
Chemical formula	C_4_Cl_2_N_2_S_2_	C_16_H_10_N_2_S_2_	C_18_H_14_N_2_O_2_S_2_	C_16_H_10_N_2_S_4_
Formula weight	211.08	294.38	354.43	358.50
Temperature (K)	100	120	120	120
Crystal system	Monoclinic	Monoclinic	Monoclinic	Orthorhombic
Space group	C2/c	P2_1_/c	P2_1_/c	Pbca
*a* (Å)	13.610(3)	7.9372 (7)	13.4370 (11)	6.0657 (3)
*b* (Å)	3.8300(7)	5.3085 (5)	3.9611 (3)	15.7108 (9)
*c* (Å)	13.843(3)	15.6078 (14)	14.6751 (12)	16.1959 (9)
*β* (°)	109.509(3)	95.2366 (18)	99.8510 (16)	90
V (Å3)	680.2(2)	654.88 (10)	769.57 (11)	1543.42 (14)
Z/Z′	4/0.5	2/0.5	2/0.5	4/0.5
d_calc_ (g cm^3^)	2.061	1.493	1.530	1.543
*µ* (Mo Kα)	14.73	8.52	8.69	8.45
2Θmax	58	58	58	58
Reflns. Collected/unique	3819/907	4430/1729	8974/2053	17,657/2047
Observed reflns [I > 2σ(I)]	822	1556	1795	1883
R_int_ (I)	0.0213	0.0213	0.0242	0.0128
R_1_ (*F*^2^)	0.0205	0.0289	0.0308	0.0245
wR_2_	0.0510	0.0794	0.0873	0.0541
GOF	1.082	1.044	1.035	1.003
Δ*ρ*_min_/Δ*ρ*_max_	−0.282/0.418	−0.202/0.407	−0.329/0.387	−0.221/0.417

## Supplementary Materials

Figures S1–S19. Crystallographic (cif files for compounds **8a** (CCDC 1840070), **8b** (CCDC 1840071), **8d** (CCDC 1840073) and **11b** (CCDC 1840072) and characterization data including ^1^H and ^13^C-NMR spectra for compounds **8a**–**f**, **11f** and **16**.
