# Supplementary material for: The Conversion of 5,5′-Bi(1,2,3-dithiazolylidenes) into Isothiazolo[5,4-d]isothiazoles
Source: Molecules. 2018 May 24;23(6):1257. doi: 10.3390/molecules23061257 (PMC6099702; doi:10.3390/molecules23061257)

Supporting Information

The Conversion of 5,5'-Bi(1,2,3-dithiazolylidenes) into Isothiazolo[5,4-*d*]isothiazoles

Lidia S. Konstantinova [1,2], Ilia V. Baranovsky [1], Vlada V. Strunyasheva [1], Andreas S. Kalogirou [3,4], Vadim V. Popov [2], Konstantin A. Lyssenko [5], Panayiotis A. Koutentis [3,*] and Oleg A. Rakitin [1,2,*]

1 N. D. Zelinsky Institute of Organic Chemistry, Russian Academy of Sciences, 119991 Moscow, Russian Federation; orakitin@ioc.ac.ru (O.A.R.); konstantinova_ls@mail.ru (L.S.K.); ilay679@rambler.ru (I.V.B.); vlada_0709@mail.ru (V.V.S.)

2 Nanotechnology Education and Research Center, South Ural State University, 454080 Chelyabinsk, Russia; rakitinoa@susu.ru (O.A.R.); popov.ioc@gmail.com (V.V.P.)

3 Department of Chemistry, University of Cyprus, P. O. Box 20537, 1678 Nicosia, Cyprus; koutenti@ucy.ac.cy (P.A.K.); kalogirou.andreas@ucy.ac.cy (A.S.K.)

4 Department of Life Sciences, School of Sciences, European University Cyprus, 6 Diogenis Str., Engomi, P. O. Box 22006, 1516 Nicosia, Cyprus; A.Kalogirou@external.euc.ac.cy (A.S.K.)

5 A. N. Nesmeyanov Institute of Organoelement Compounds, Russian Academy of Sciences, 119991 Moscow, Russia; kostya@ineos.ac.ru

* Correspondence: koutenti@ucy.ac.cy; Tel.: +357 22 892783; orakitin@ioc.ac.ru; Tel.: +7 499 135 5327

Contents **Page**

S1. Crystallographic Data **S3**

S2. ^{1}H- and ^{13}C-NMR Spectra of Compounds 8a-f, 11f and 16 **S6**

S1. Crystallographic Data

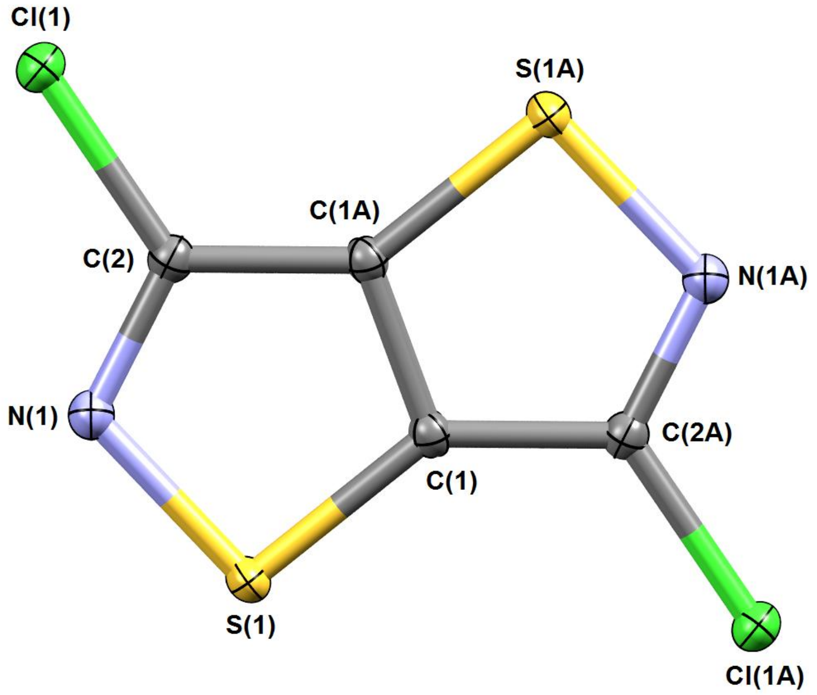

Figure S1. X-Ray structure of 3,6-dichloroisothiazolo[5,4-*d*]isothiazole (**8a**). (CCDC 1840070). Thermal ellipsoids are at 50% probability.

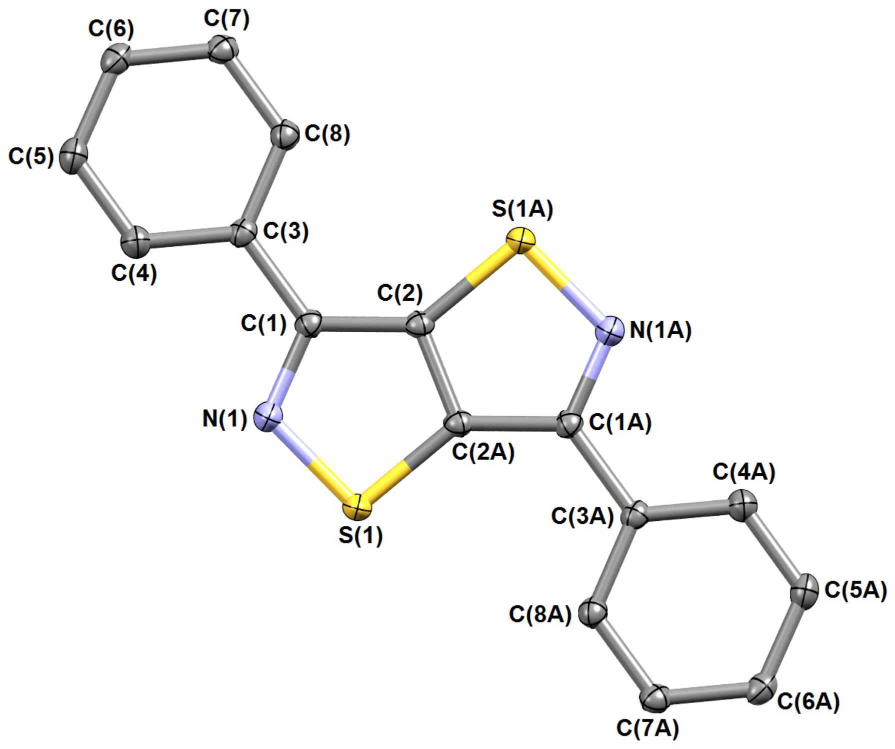

Figure S2. X-Ray structure of 3,6-diphenylisothiazolo[5,4-d]isothiazole (8b) (CCDC 1840071). Thermal ellipsoids are at 50% probability and hydrogens are omitted for clarity.

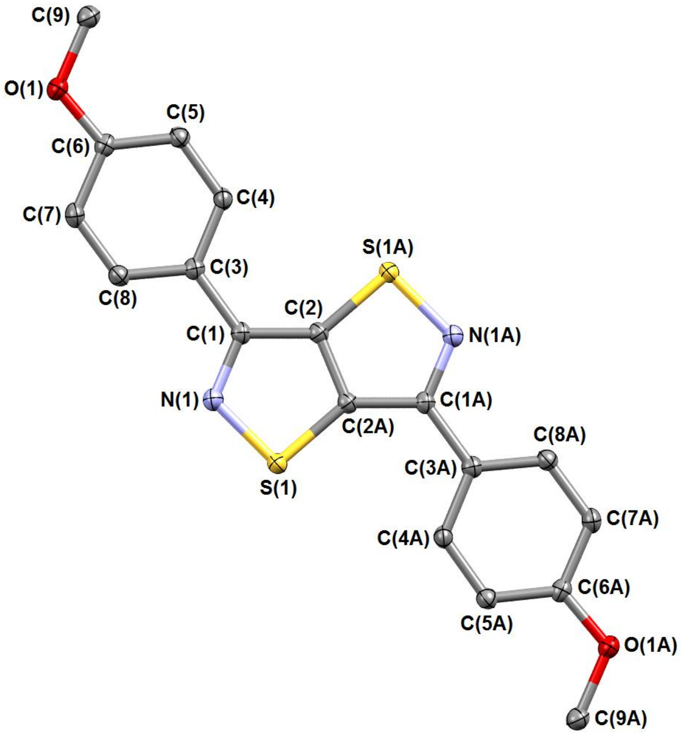

Figure S3. X-Ray structure of 3,6-bis(4-methoxyphenyl)isothiazolo[5,4-d]isothiazole (8d) (CCDC 1840073). Thermal ellipsoids are at 50% probability and hydrogens are omitted for clarity.

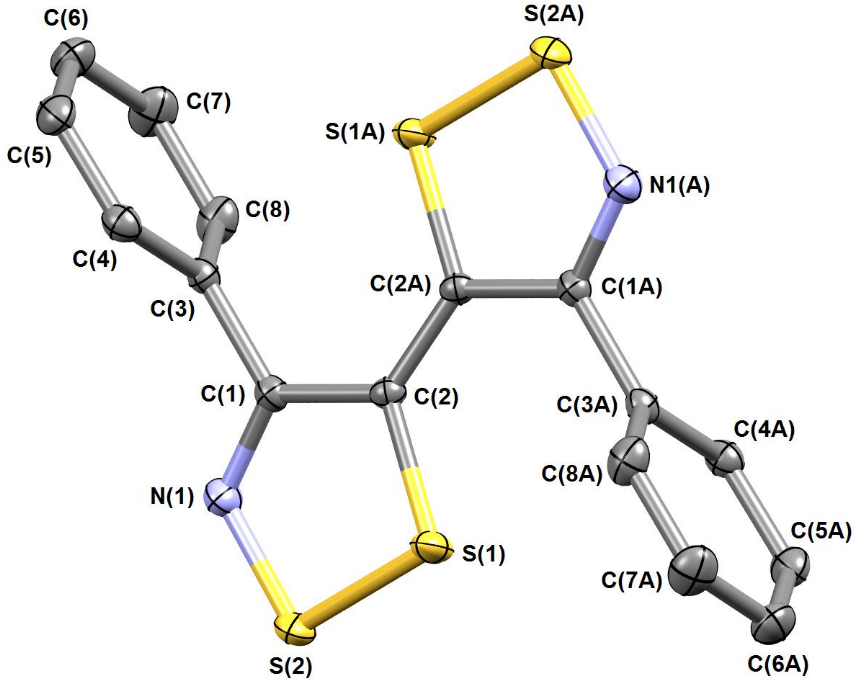

Figure S4. X-Ray structure of (E)-4,4'-diphenyl-5,5'-bi(1,2,3-dithiazolylidene) (11b) (CCDC 1840072). Thermal ellipsoids are at 50% probability and hydrogens are omitted for clarity.

S2. ^{1}H- and ^{13}C-NMR Spectra of Compounds 8a-f, 11f and 16

Figure S5. 13C-NMR spectrum of 3,6-dichloroisothiazolo[5,4-d]isothiazole (8a) (75 MHz, CDCl3)

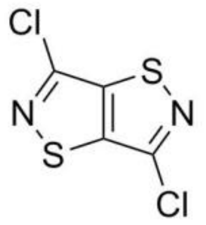

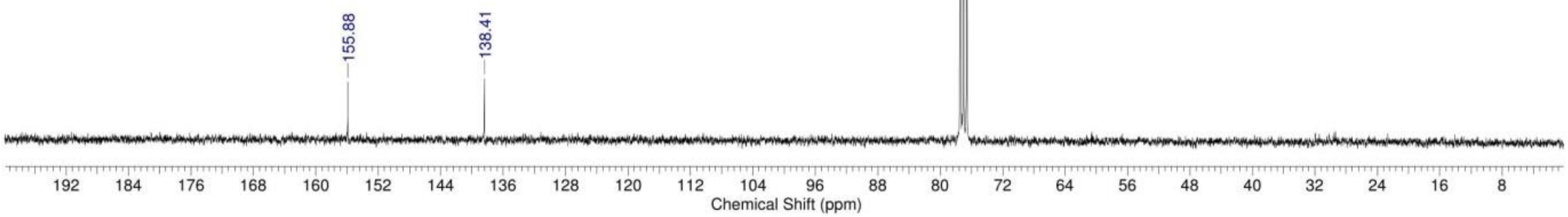

Figure S6. 1H-NMR spectrum of 3,6-diphenylisothiazolo[5,4-d]isothiazole (8b) (300 MHz, CDCl3)

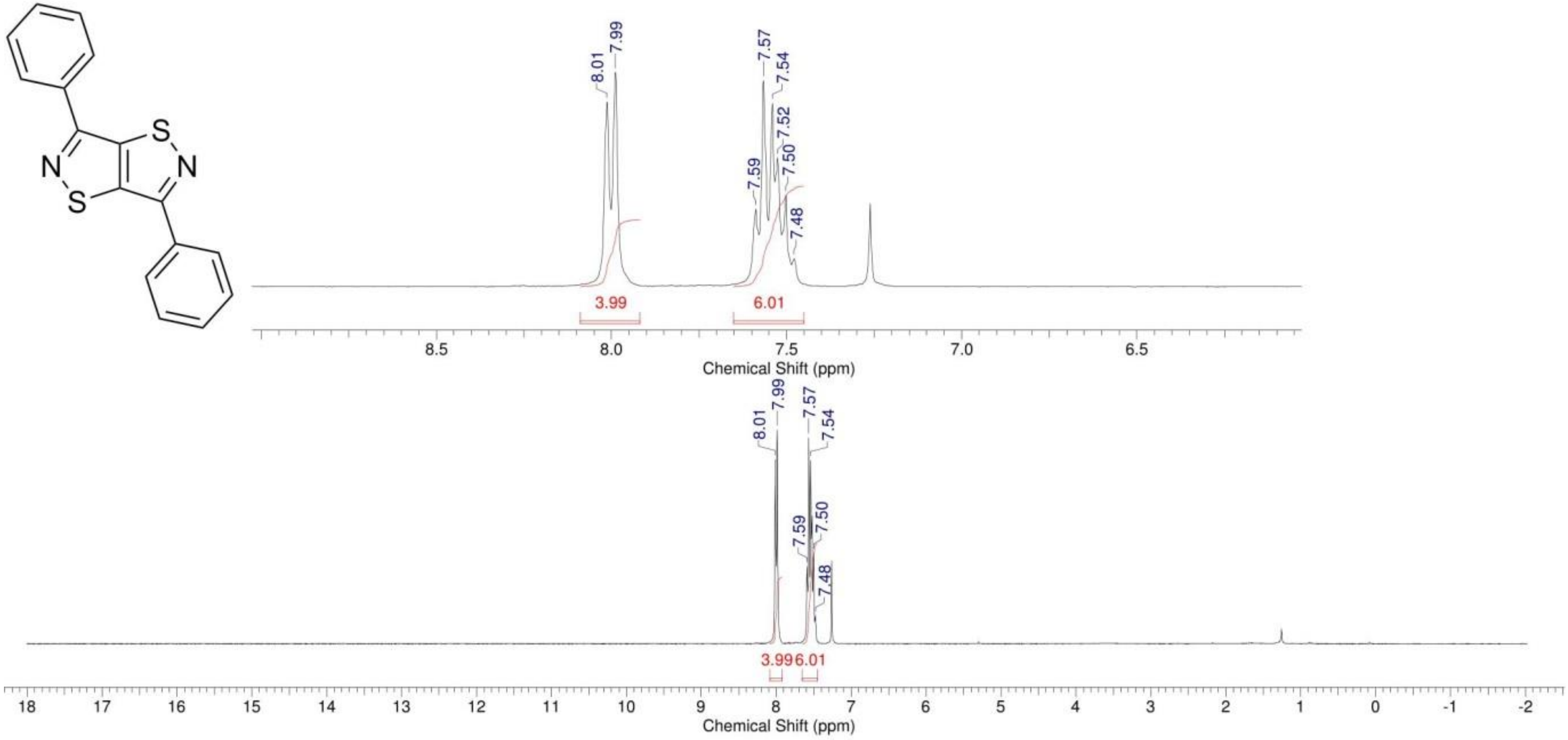

Figure S7. 13C-NMR spectrum of 3,6-diphenylisothiazolo[5,4-d]isothiazole (8b) (75 MHz, CDCl3)

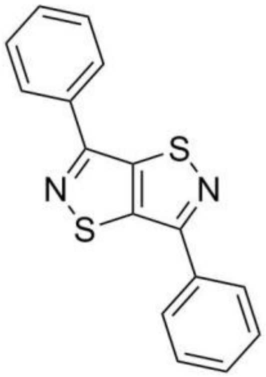

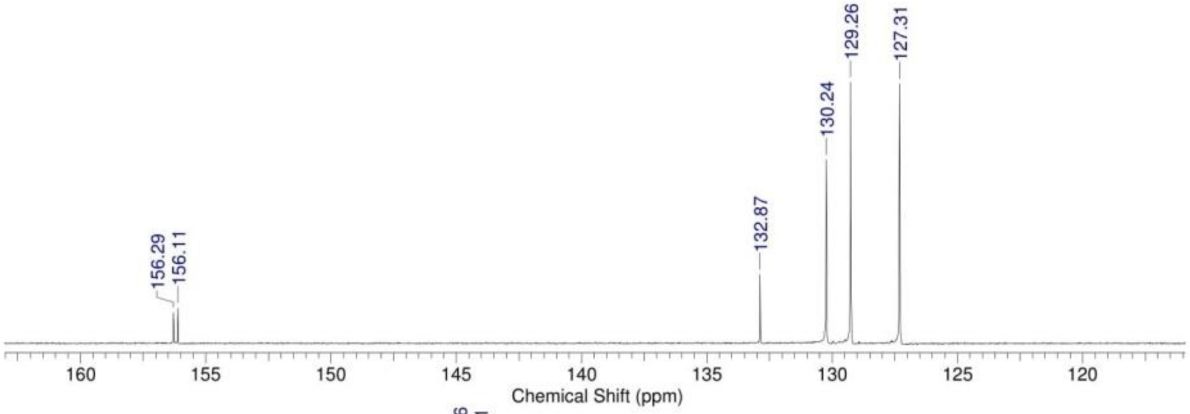

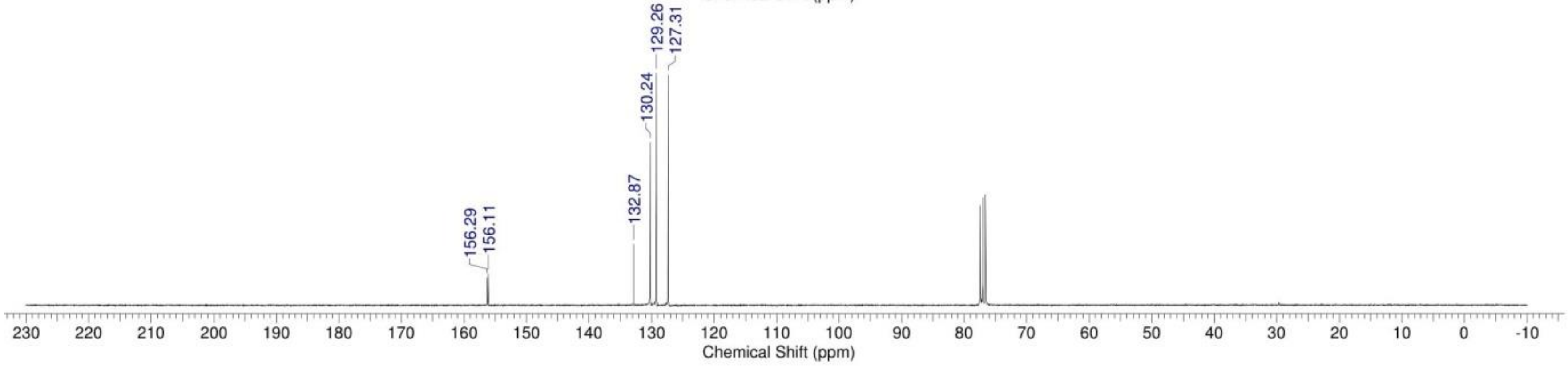

Figure S8. 1H-NMR spectrum of 3,6-bis(4-fluorophenyl)isothiazolo[5,4-d]isothiazole (8c) (300 MHz, DMSO-d6)

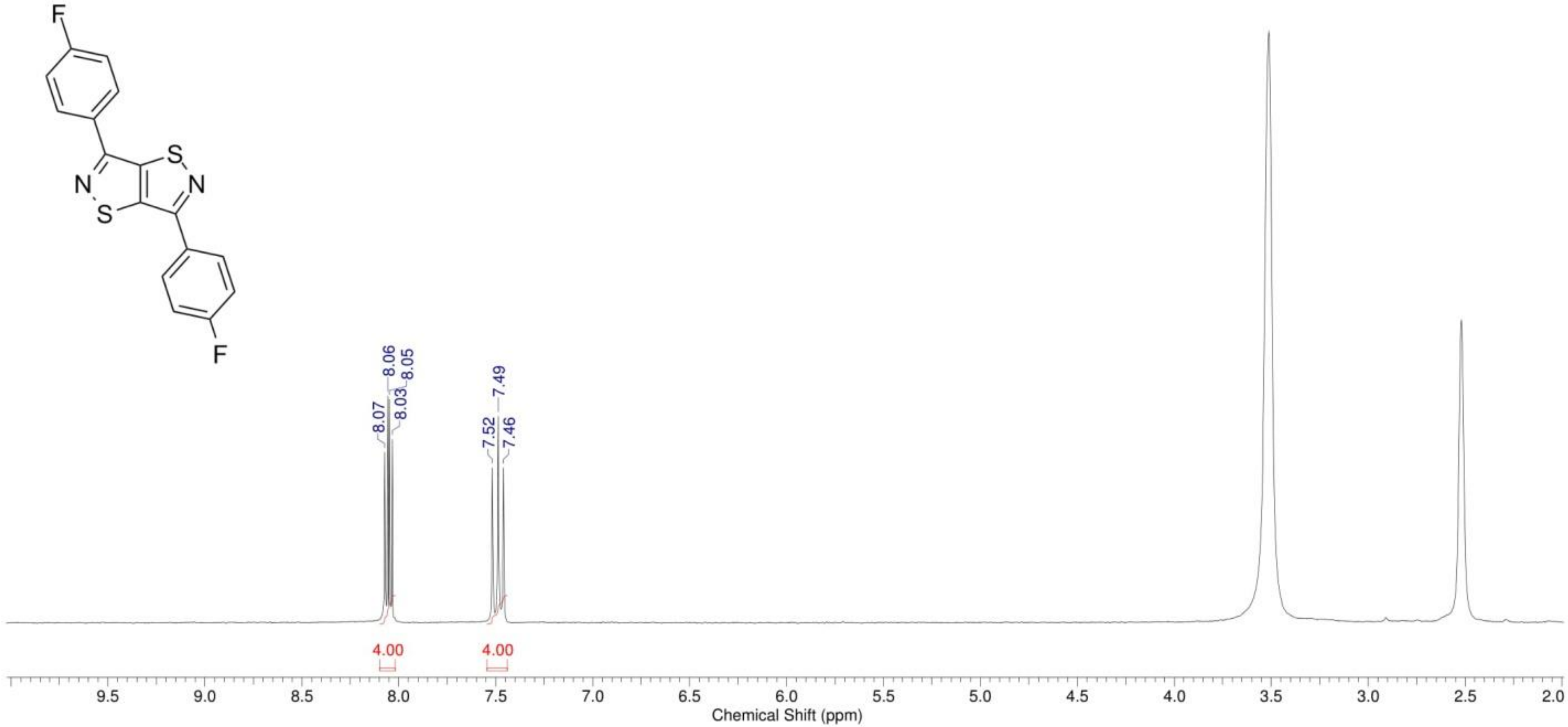

Figure S9. 13C-NMR spectrum of 3,6-bis(4-fluorophenyl)isothiazolo[5,4-d]isothiazole (8c) (150 MHz, DMSO-d6)

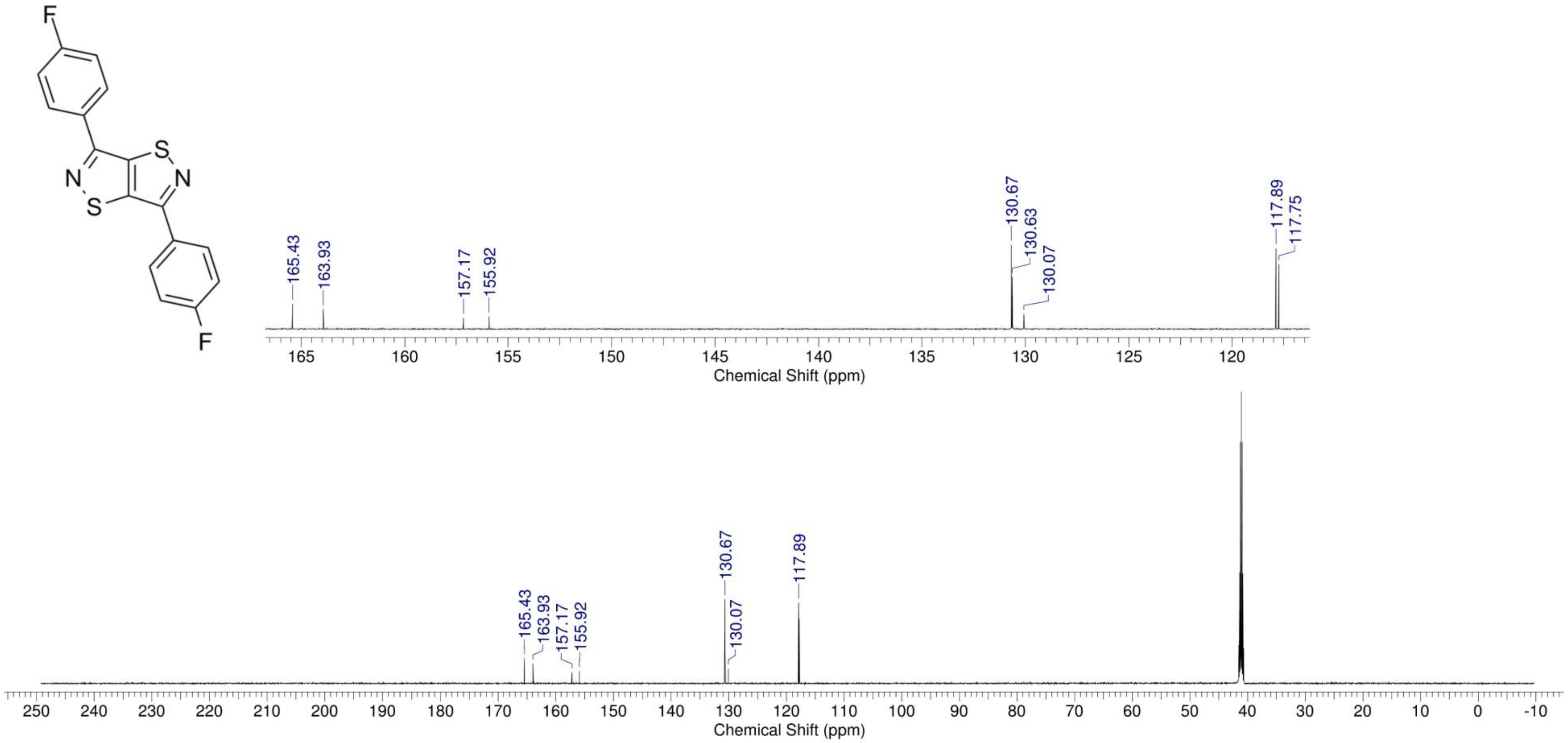

Figure S10. 1H-NMR spectrum of 3,6-bis(4-methoxyphenyl)isothiazolo[5,4-d]isothiazole (8d) (300 MHz, DMSO-d6)

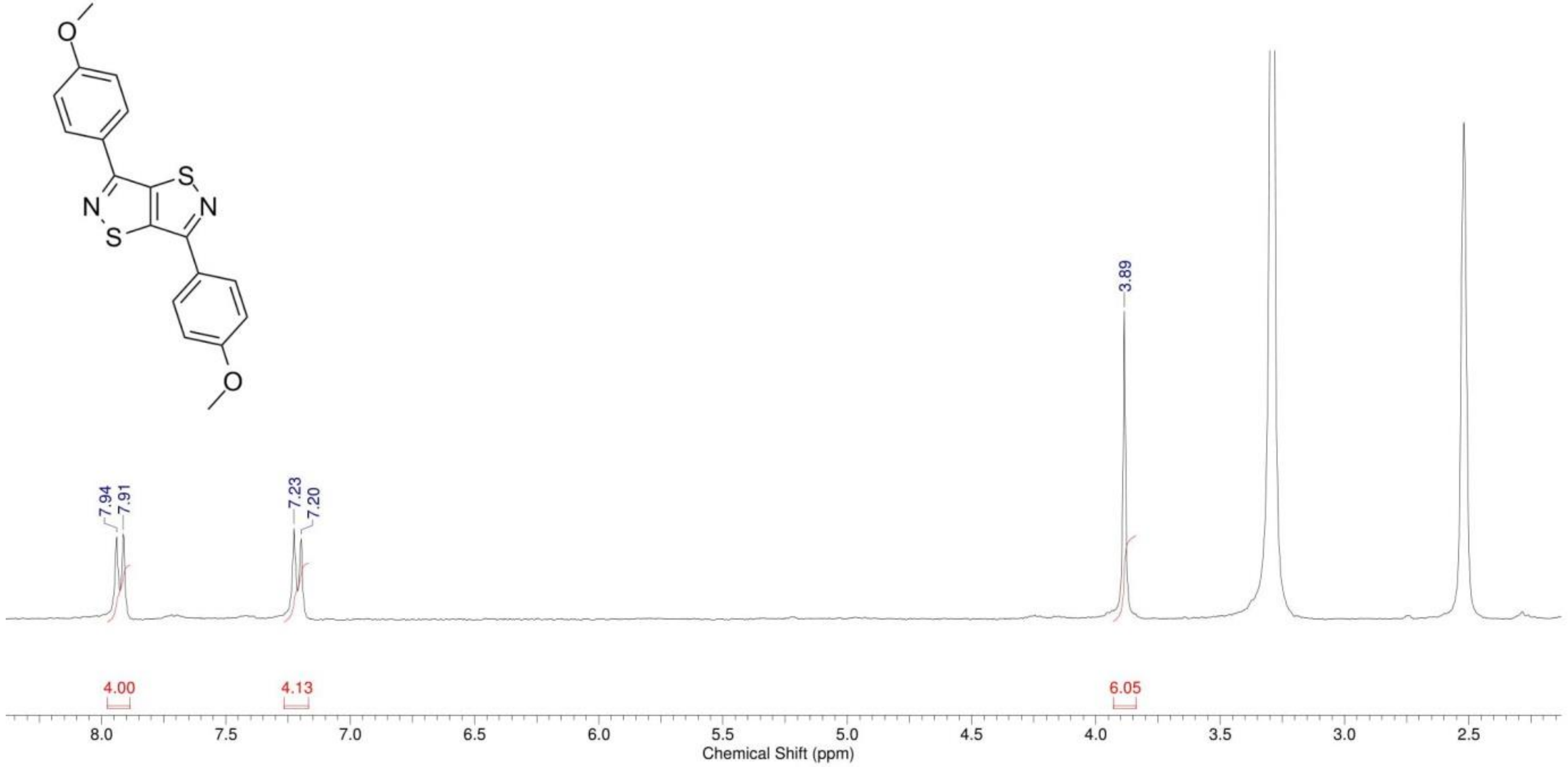

Figure S11. 13C-NMR spectrum of 3,6-bis(4-methoxyphenyl)isothiazolo[5,4-d]isothiazole (8d) (150 MHz, DMSO-d6)

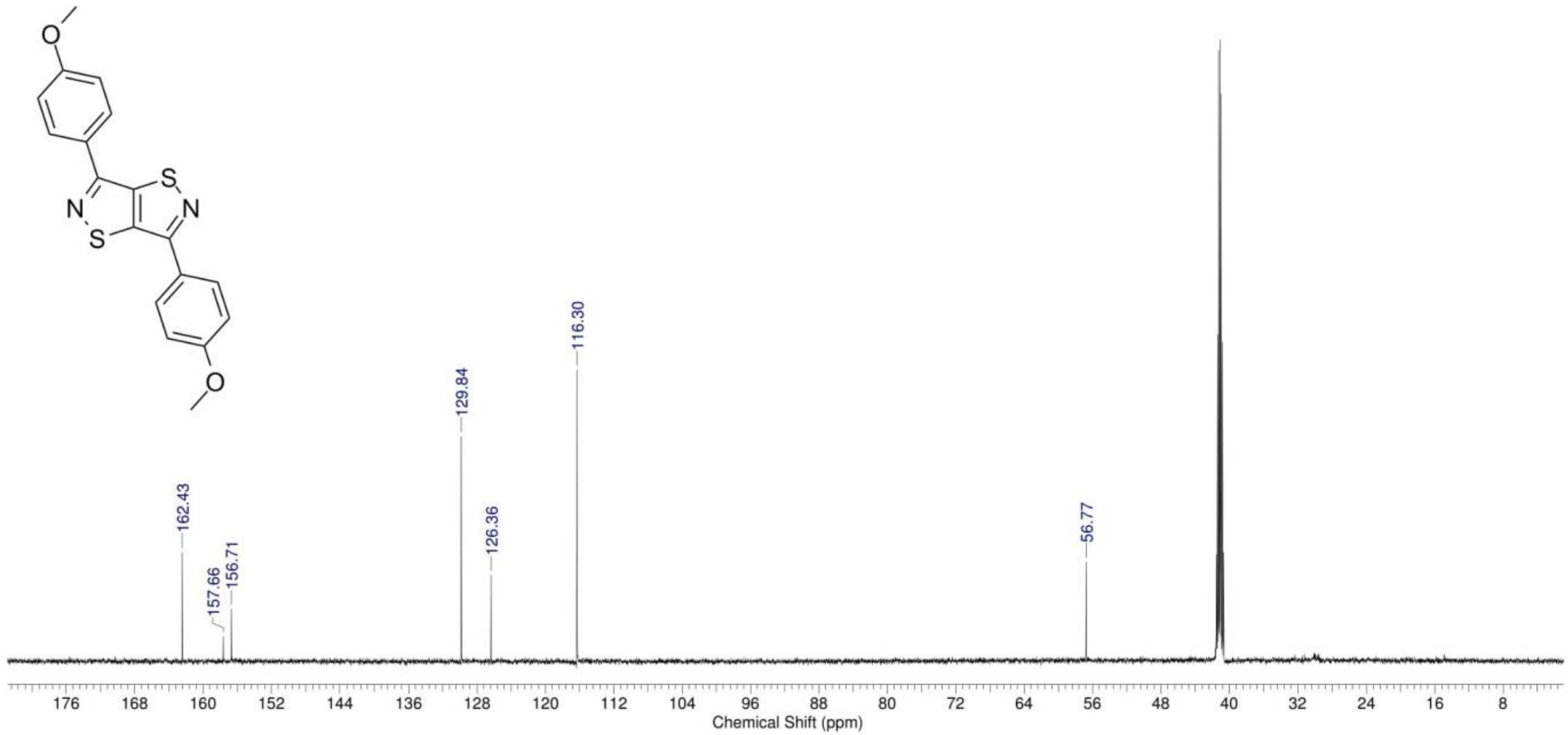

Figure S12. 1H-NMR spectrum of 3,6-di(thien-2-yl)isothiazolo[5,4-d]isothiazole (8e) (300 MHz, CDCl3)

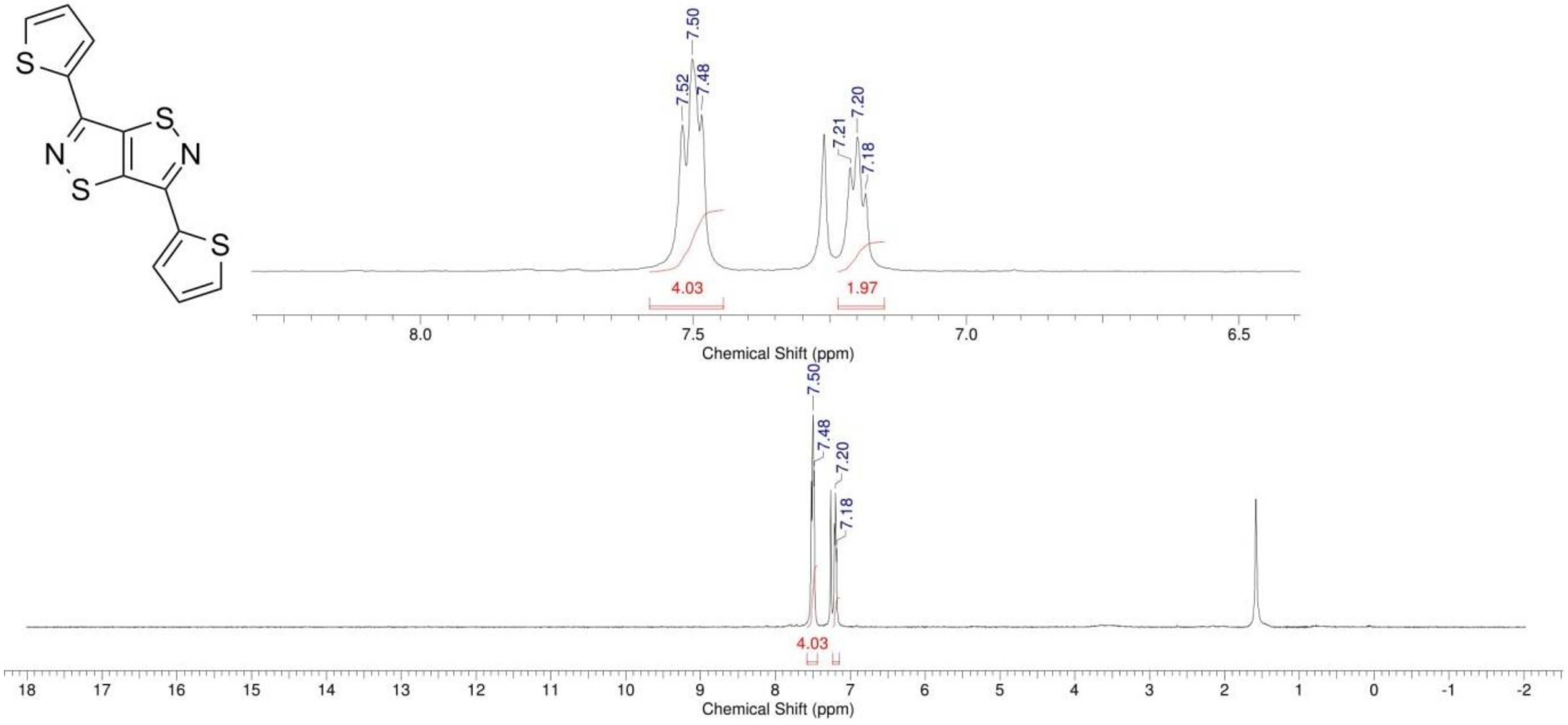

Figure S13. 13C-NMR spectrum of 3,6-di(thien-2-yl)isothiazolo[5,4-d]isothiazole (8e) (75 MHz, CDCl3)

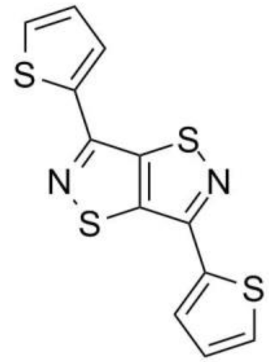

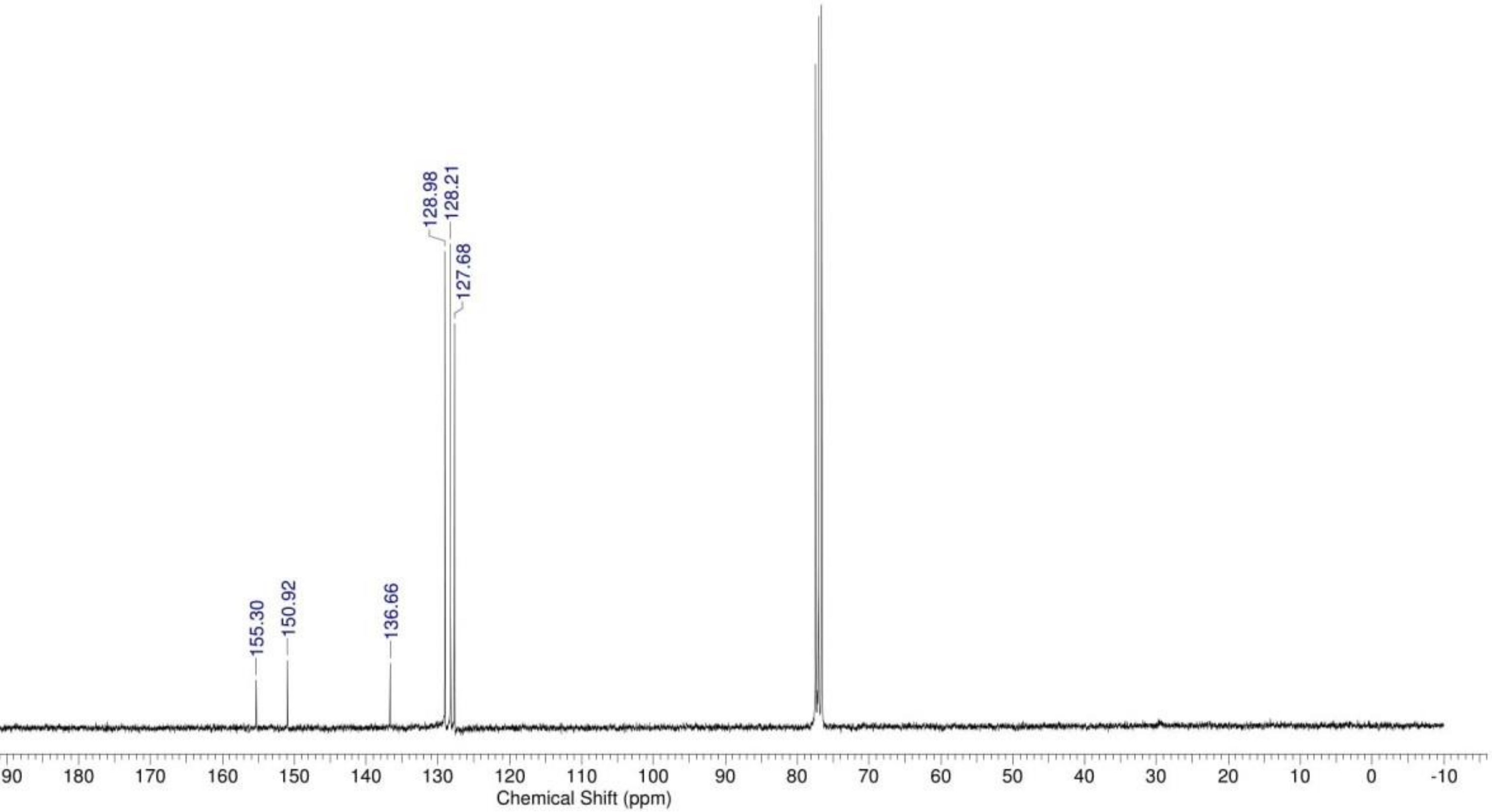

Figure S14. 1H-NMR spectrum of 3,6-bis(4-bromophenyl)isothiazolo[5,4-d]isothiazole (8f) (300 MHz, CD2Cl2)

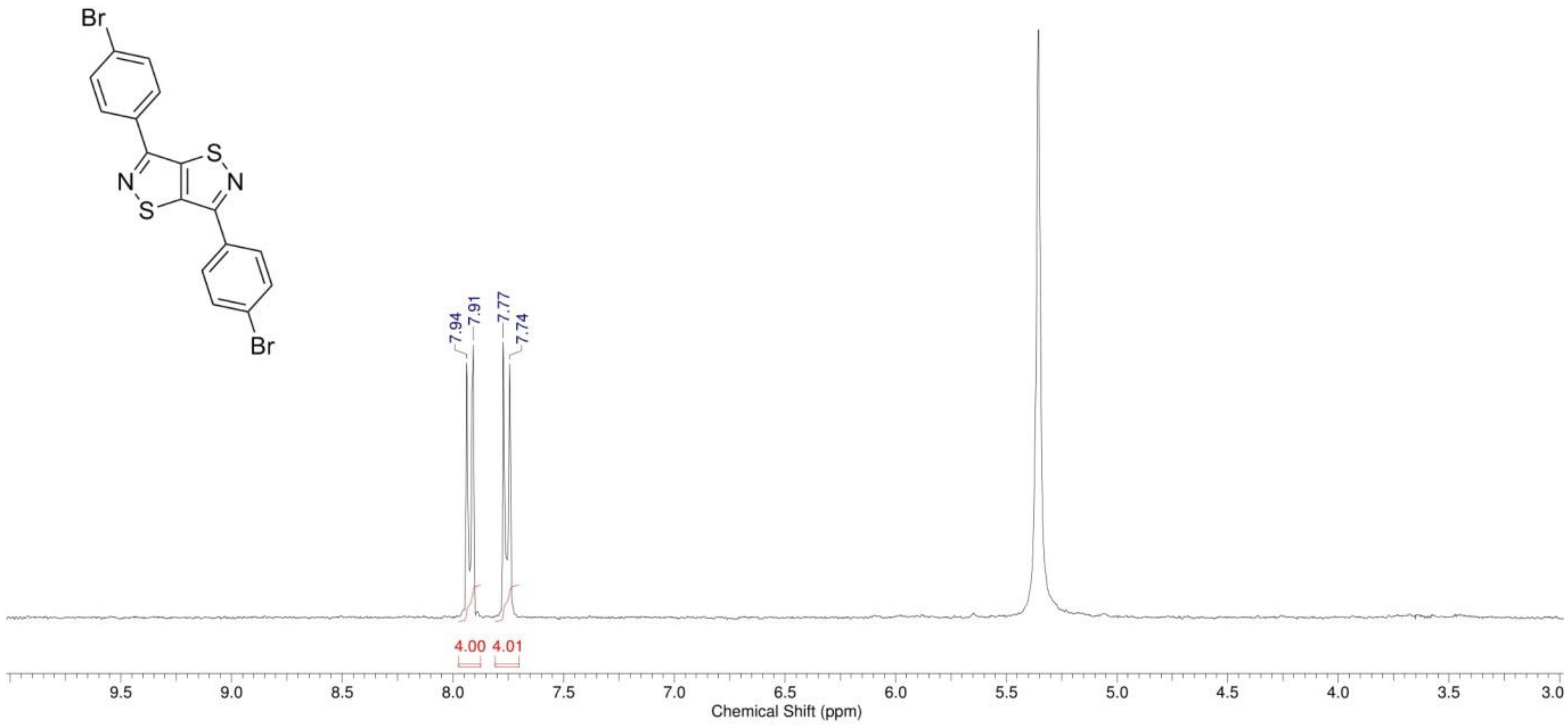

Figure S15. ^{13}C-NMR spectrum of 3,6-bis(4-bromophenyl)isothiazolo[5,4-*d*]isothiazole (**8f**) (125 MHz, CD_2Cl_2)

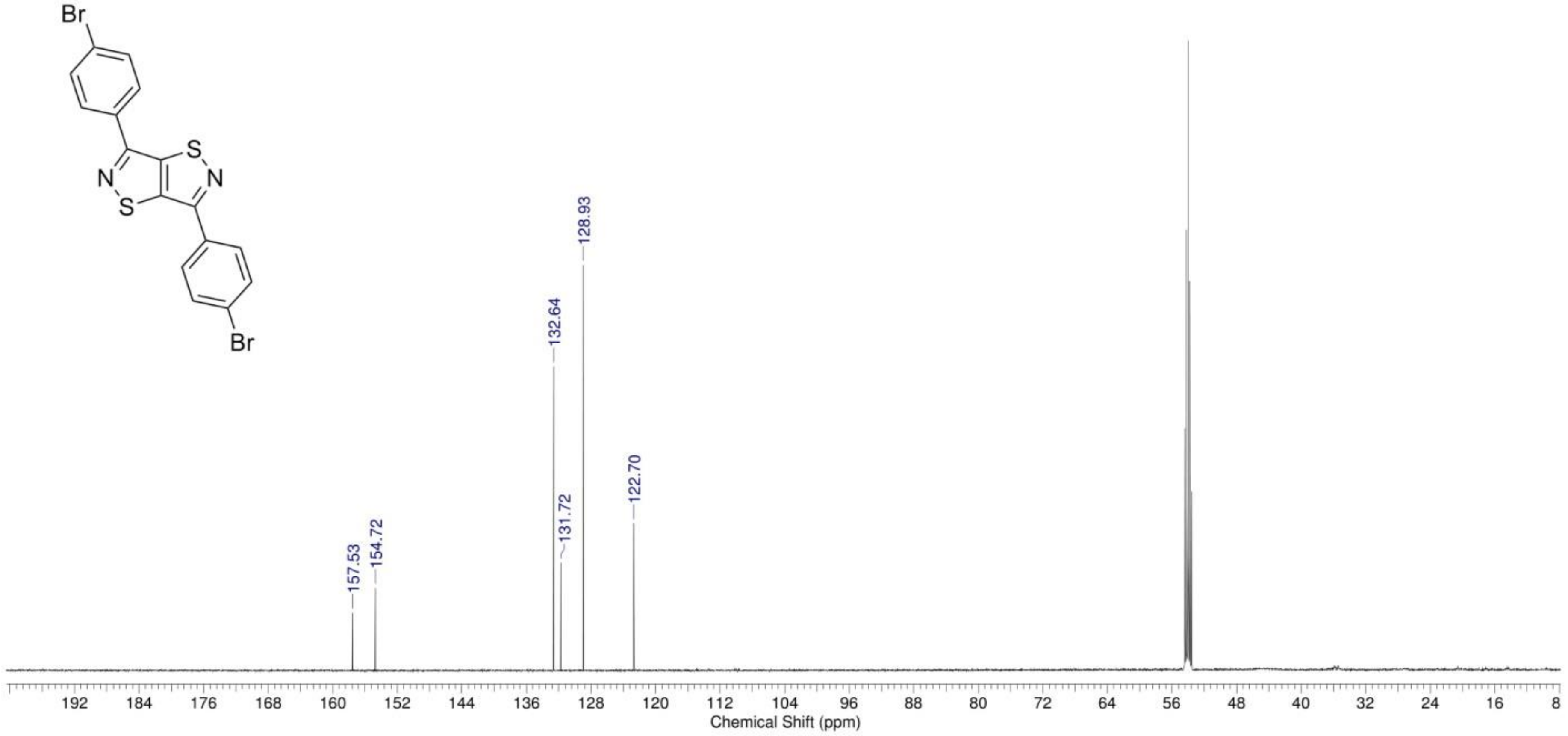

Figure S16. ^{1}H-NMR spectrum of 3,6-di(5,5'-dibromothien-2-yl)isothiazolo[5,4-*d*]isothiazole (**16**) (300 MHz, D_2SO_4)

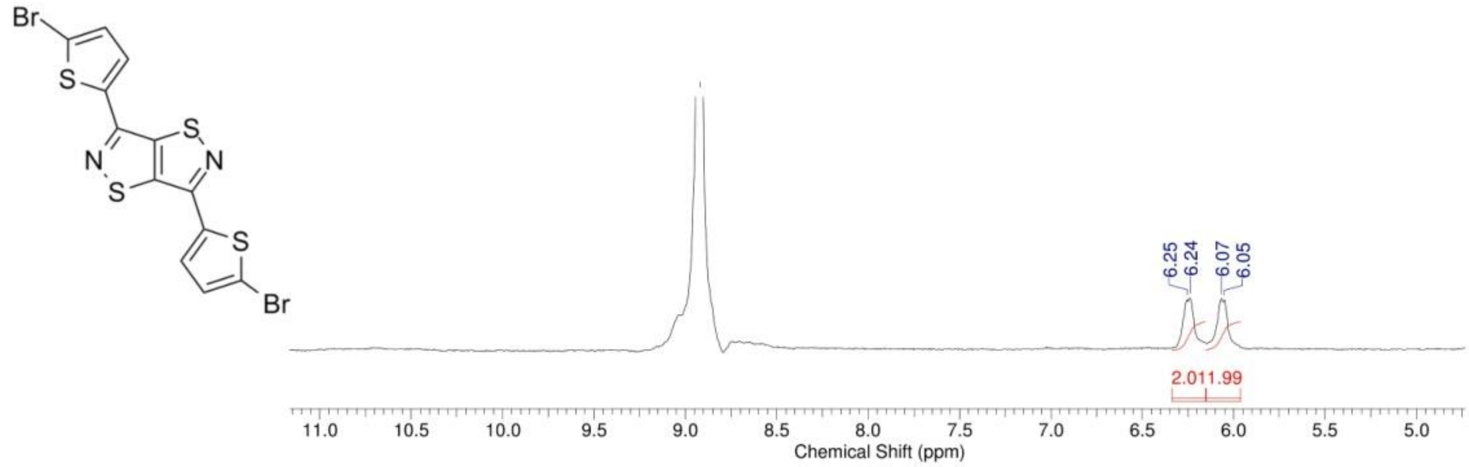

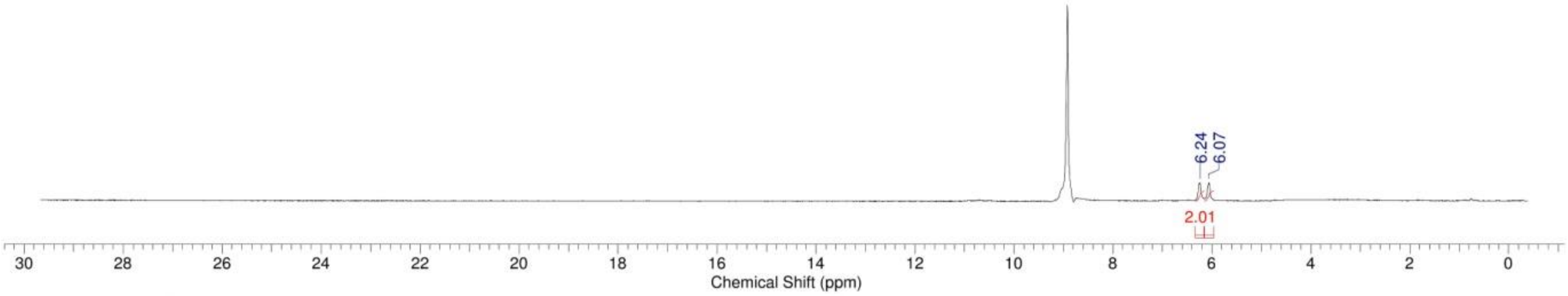

Figure S17. ^{13}C-NMR spectrum of 3,6-di(5,5'-dibromothien-2-yl)isothiazolo[5,4-*d*]isothiazole (**16**) (125 MHz, D_2SO_4)

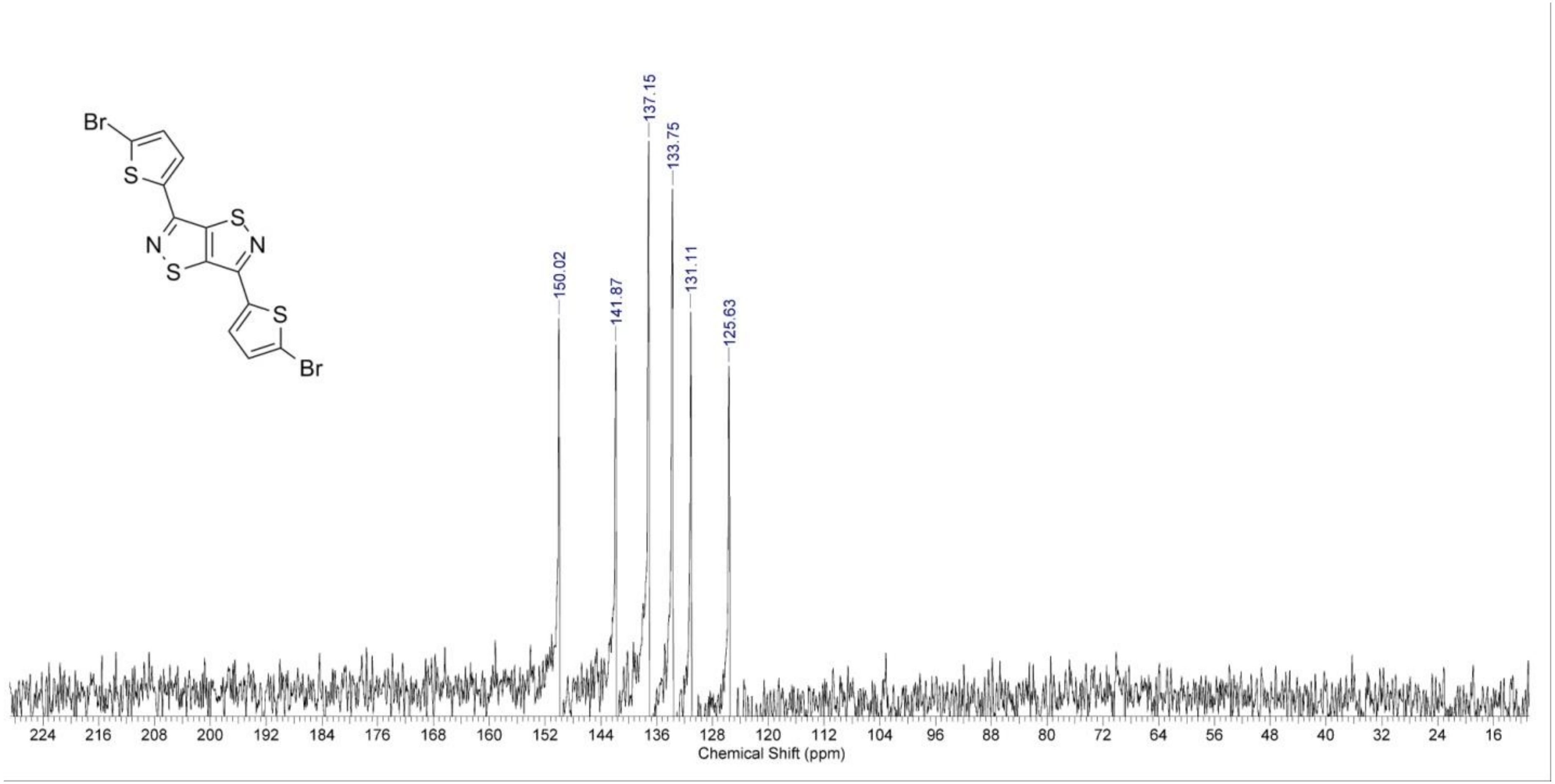

Figure S18. ^{1}H-NMR spectrum of 4,4'-bis(4-bromophenyl)-5,5'-bi-1,2,3-dithiazole (**11f**) (300 MHz, CD_2Cl_2)

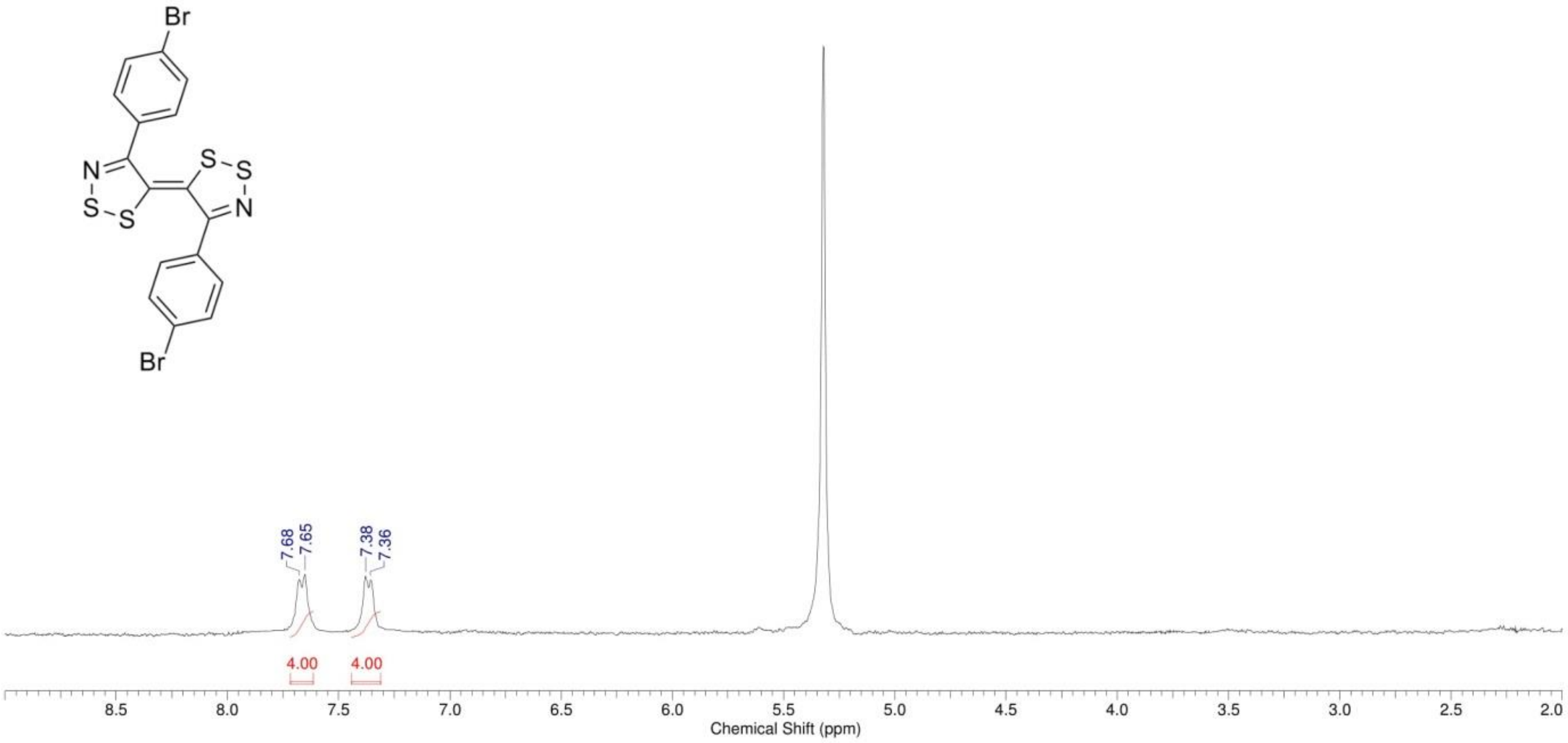

Figure S19. ^{13}C-NMR spectrum of 4,4'-bis(4-bromophenyl)-5,5'-bi-1,2,3-dithiazole (**11f**) (75 MHz, CD_2Cl_2)

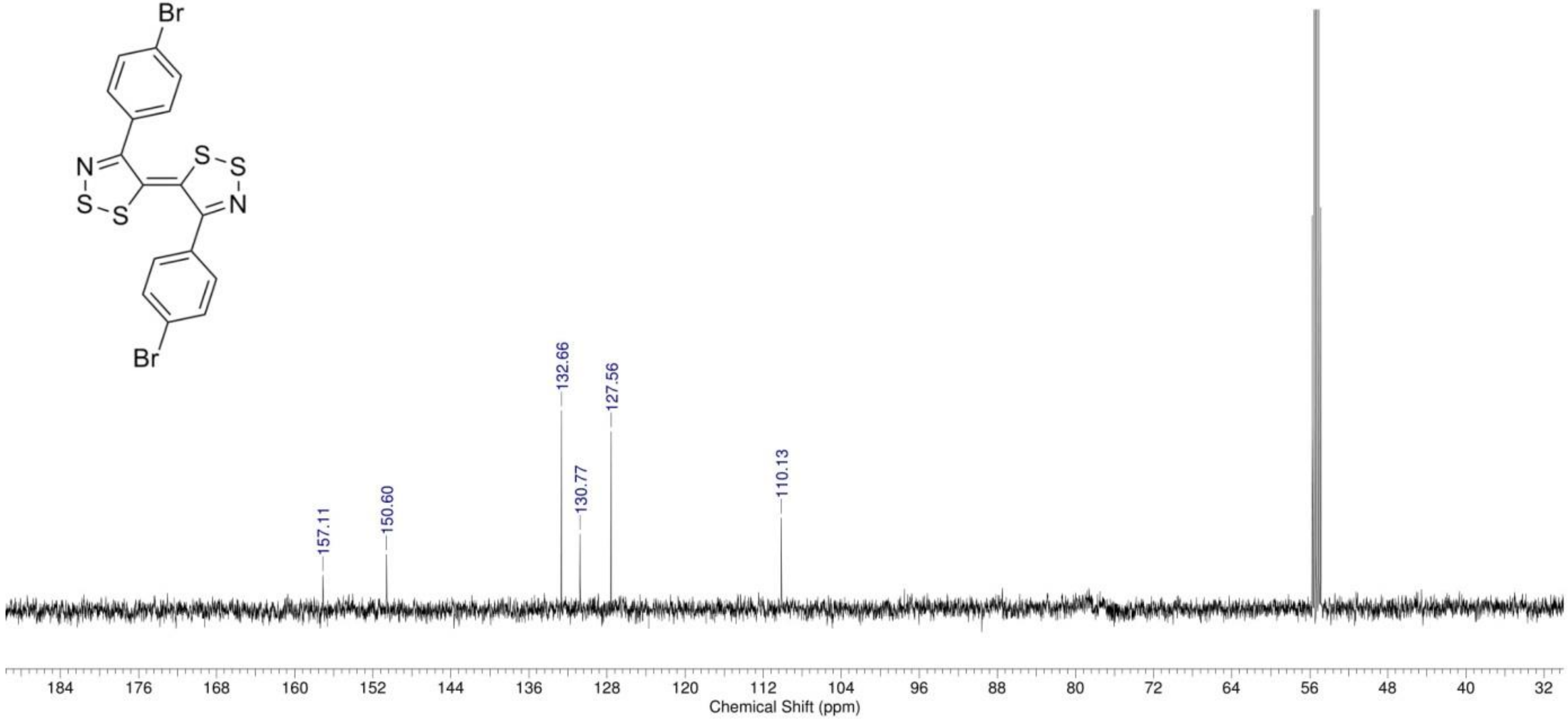

Supplement: Supplementary file 1 [file molecules-23-01257-s001.pdf]
